# Supplementary figures and images for: Basal MET phosphorylation is an indicator of hepatocyte dysregulation in liver disease
Source: Mol Syst Biol. 2024 Jan 12;20(3):187–216. doi: 10.1038/s44320-023-00007-4 (PMC10912216; doi:10.1038/s44320-023-00007-4)

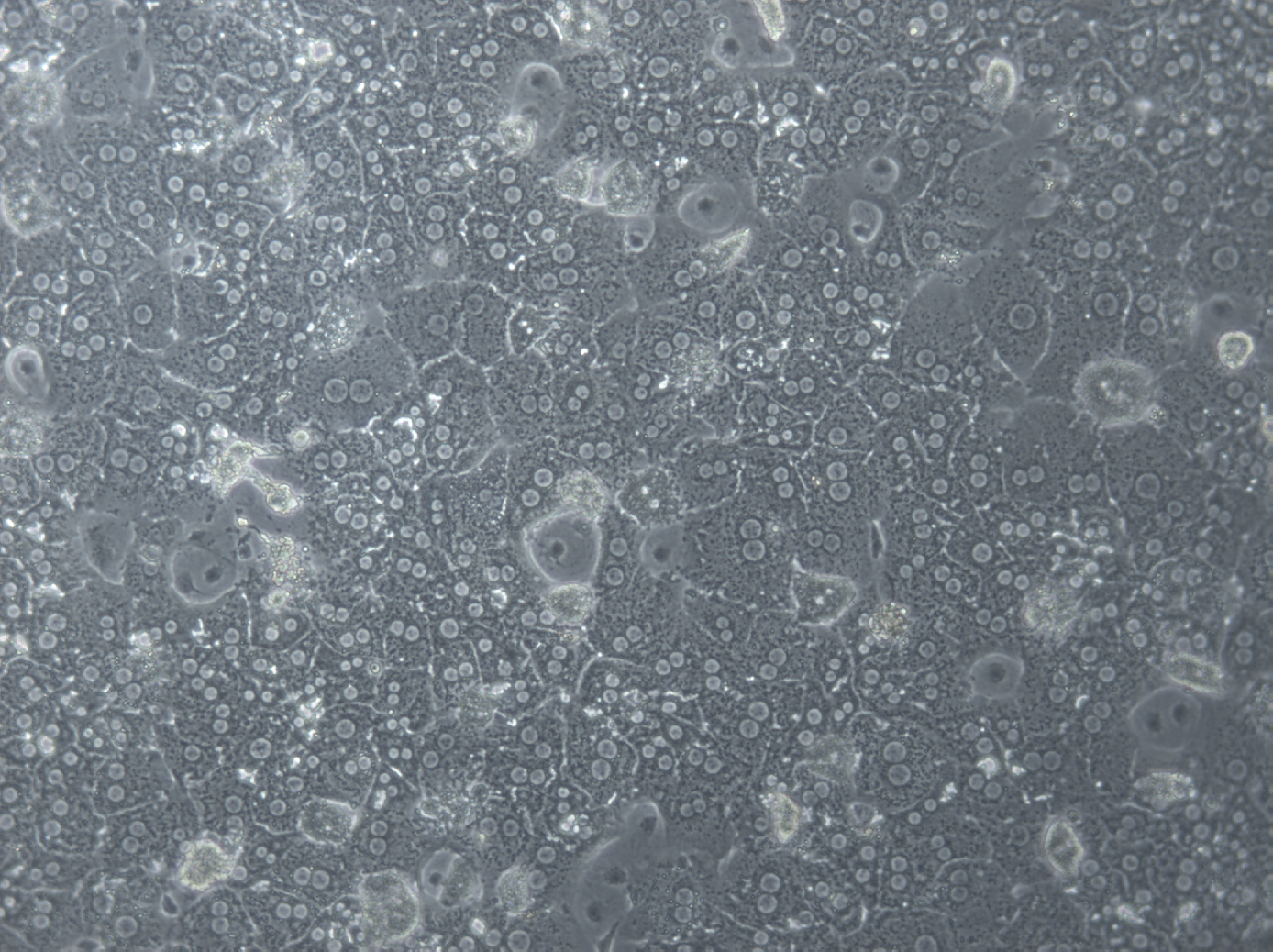

Supplement: Supplementary file 8 — Source Data Fig. 1 [file 44320_2023_7_MOESM8_ESM.zip › Figure 1/1C/PMH_SD.tiff]

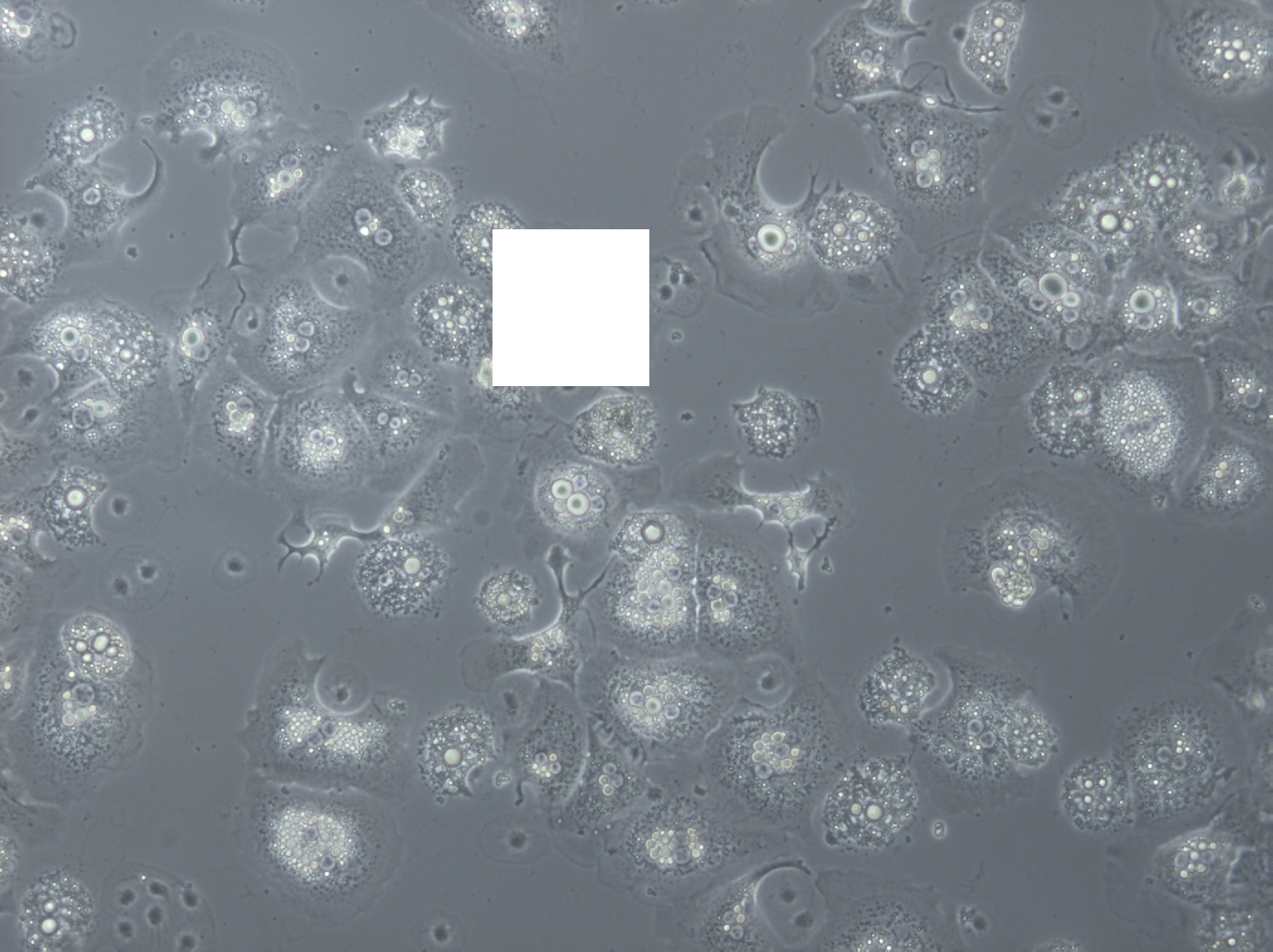

Supplement: Supplementary file 8 — Source Data Fig. 1 [file 44320_2023_7_MOESM8_ESM.zip › Figure 1/1C/PMH_WD.tif]

\* spilled in 2 lanes

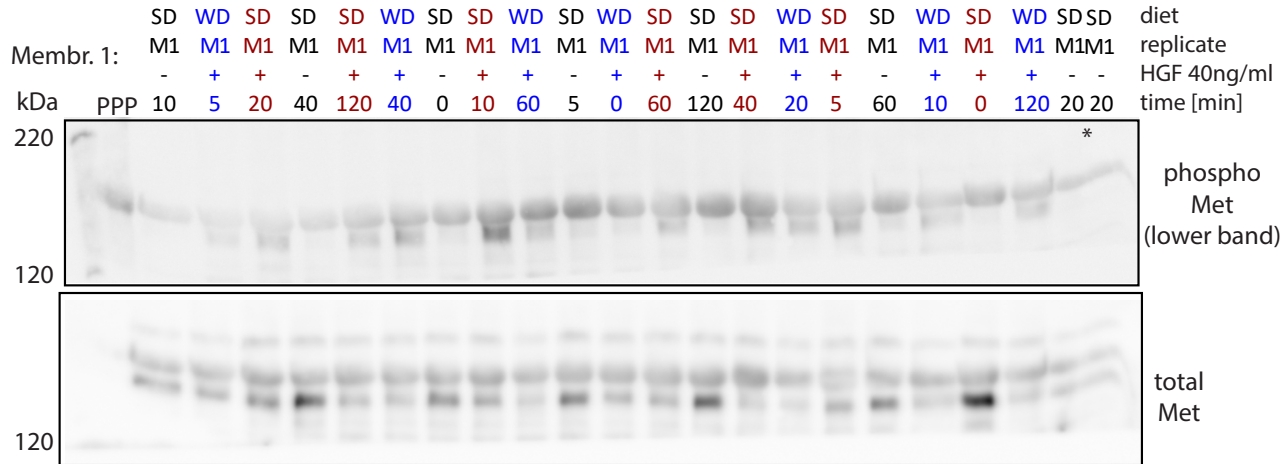

Supplement: Supplementary file 9 — Source Data Fig. 2 [file 44320_2023_7_MOESM9_ESM.zip › Figure 2/2C/Gel1-2_B1_pMet_tMet.pdf]

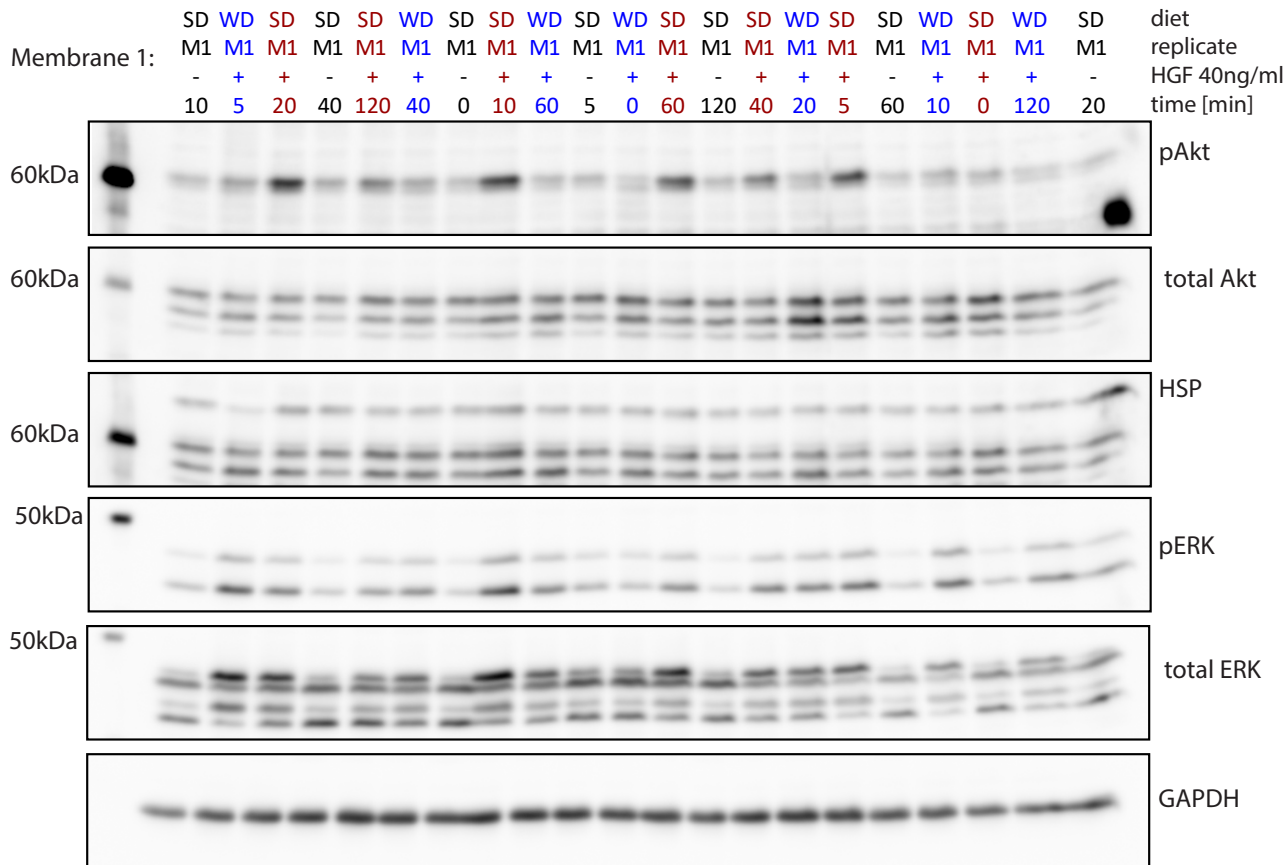

Supplement: Supplementary file 9 — Source Data Fig. 2 [file 44320_2023_7_MOESM9_ESM.zip › Figure 2/2C/Gel1_B1_pAkt_tAkt_pERK_tERK.pdf]

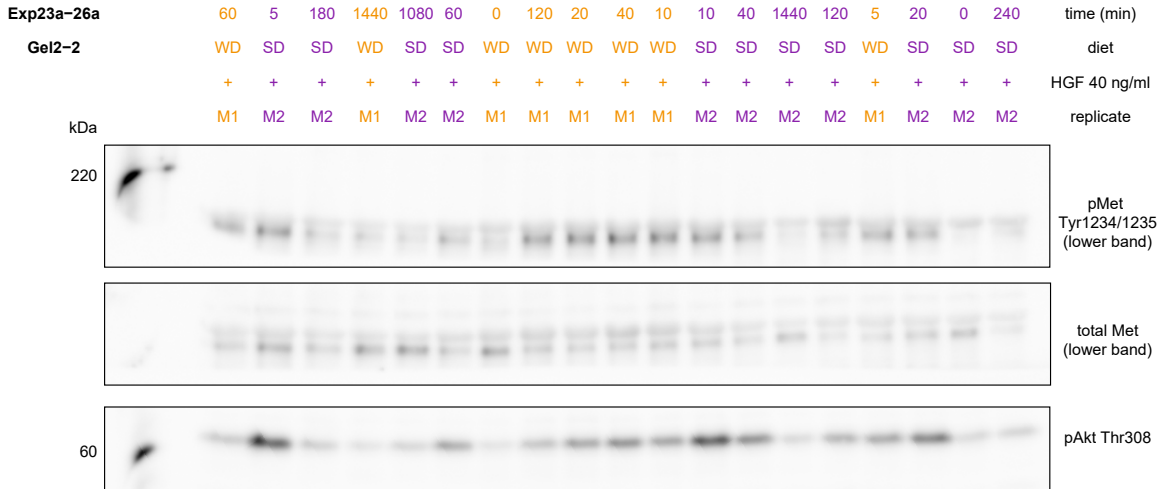

Supplement: Supplementary file 9 — Source Data Fig. 2 [file 44320_2023_7_MOESM9_ESM.zip › Figure 2/2C/Gel2-2_B3a_pMet_tMet_pAktT308.pdf]

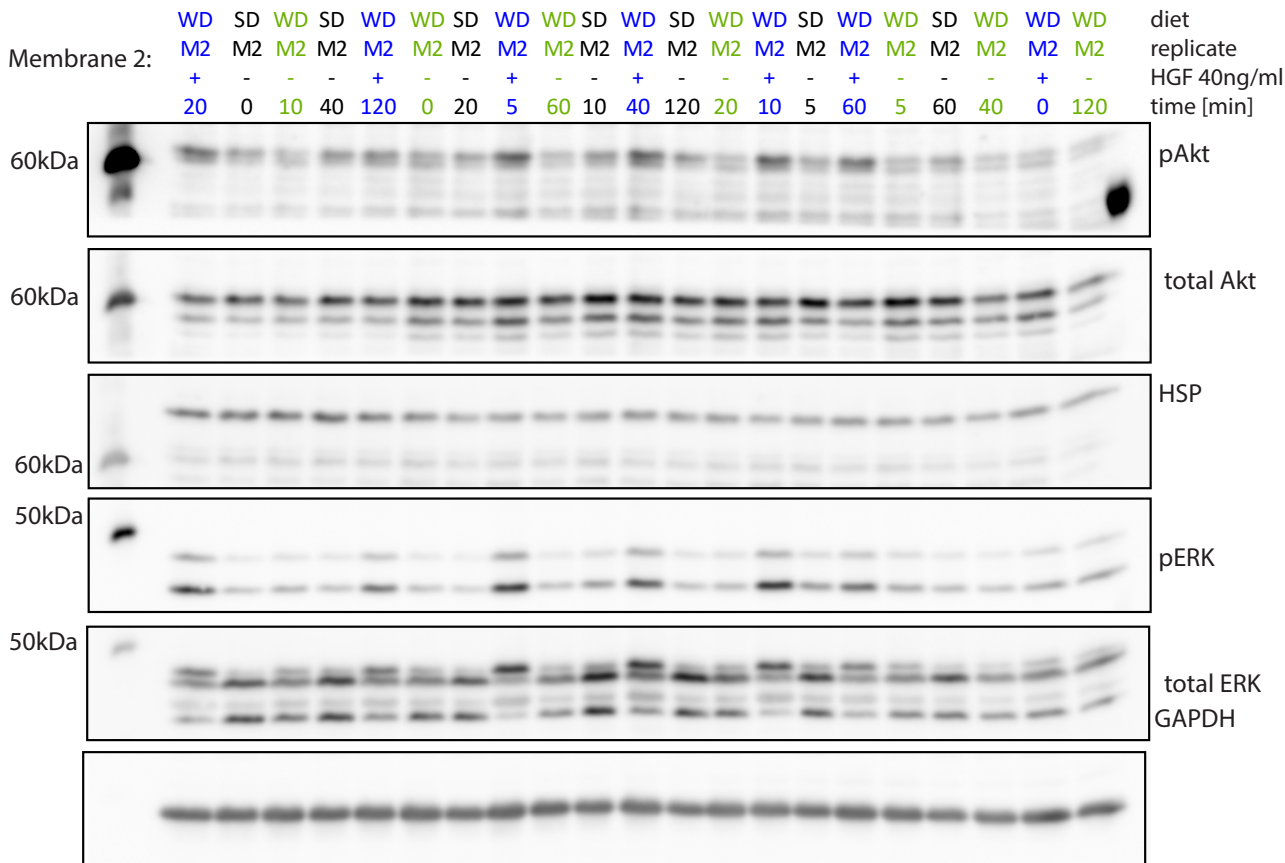

Supplement: Supplementary file 9 — Source Data Fig. 2 [file 44320_2023_7_MOESM9_ESM.zip › Figure 2/2C/Gel2_B1_pAkt_tAkt_pERK_tERK.pdf]

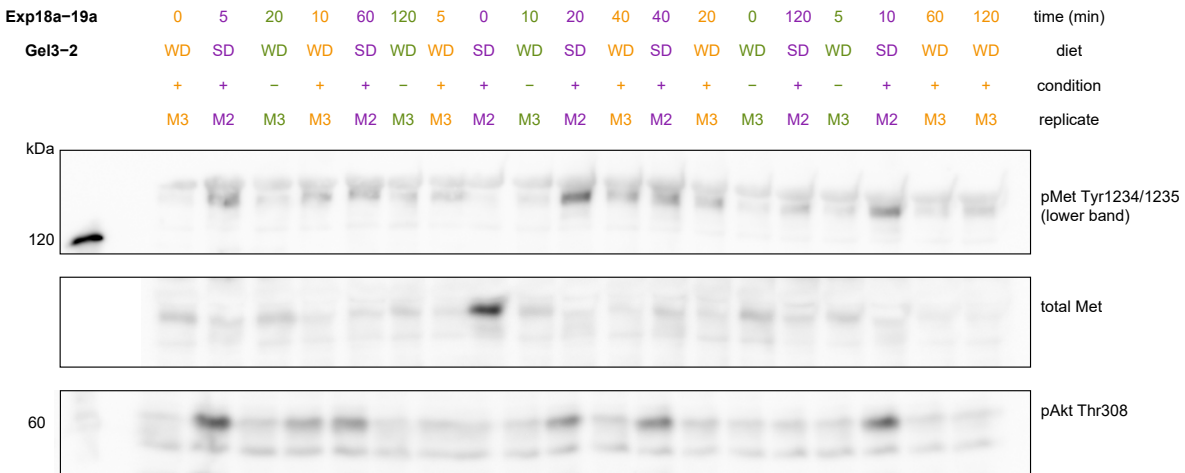

Supplement: Supplementary file 9 — Source Data Fig. 2 [file 44320_2023_7_MOESM9_ESM.zip › Figure 2/2C/Gel3-2_B1_pMet_tMet_pAktT308.pdf]

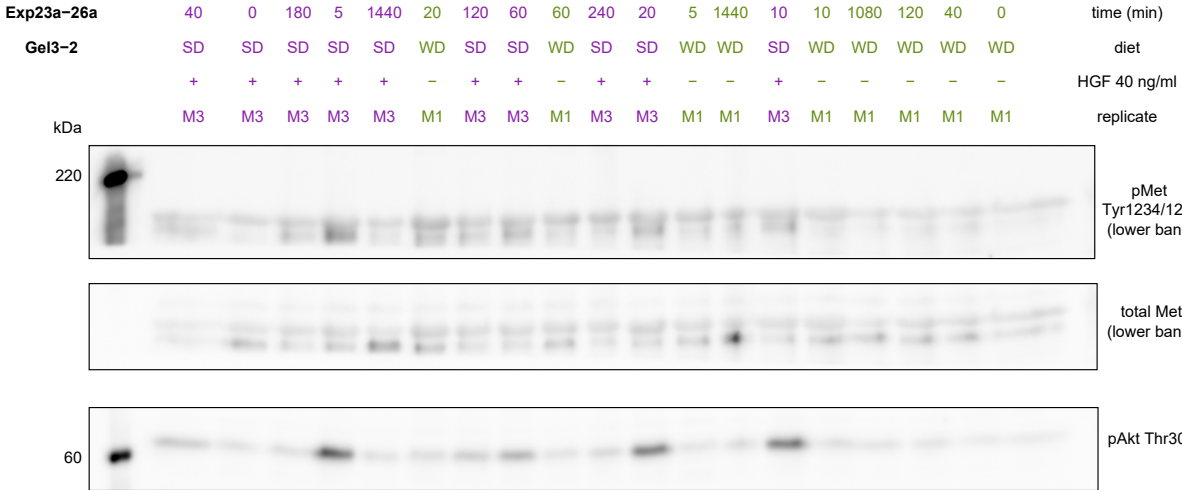

Supplement: Supplementary file 9 — Source Data Fig. 2 [file 44320_2023_7_MOESM9_ESM.zip › Figure 2/2C/Gel3-2_B3a_pMet_tMet_pAktT308.pdf]

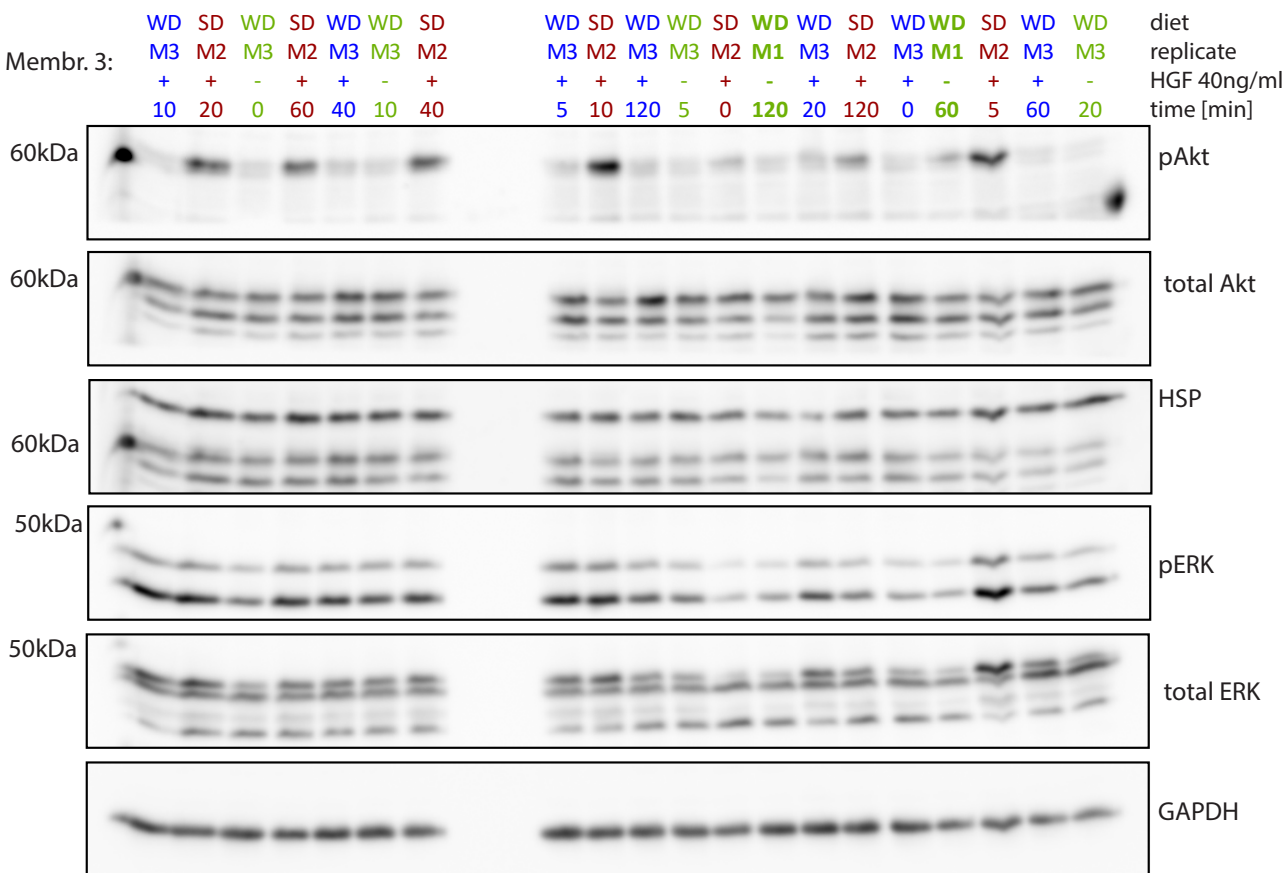

Supplement: Supplementary file 9 — Source Data Fig. 2 [file 44320_2023_7_MOESM9_ESM.zip › Figure 2/2C/Gel3_B1_pAkt_tAkt_pERK_tERK.pdf]

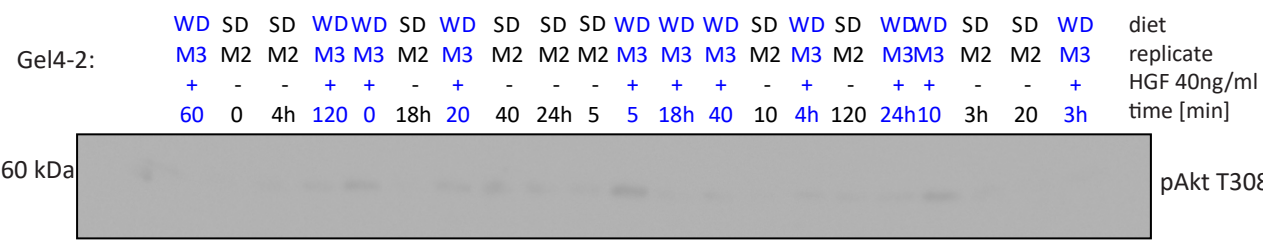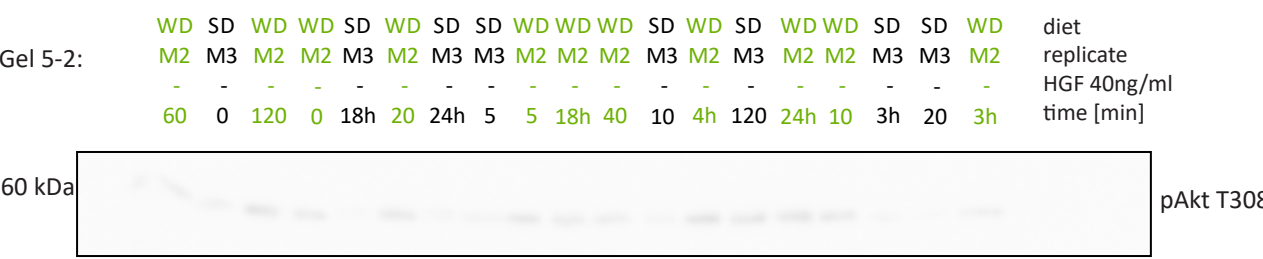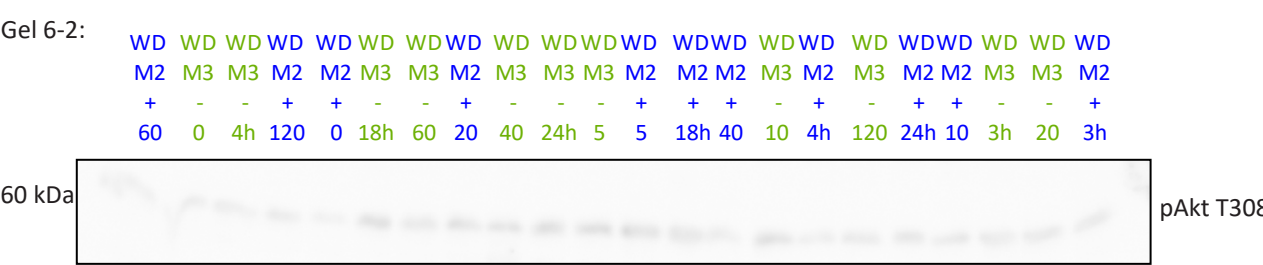

Supplement: Supplementary file 9 — Source Data Fig. 2 [file 44320_2023_7_MOESM9_ESM.zip › Figure 2/2C/Gel4-2_5-2_6-2_B2_pAktT308.pdf]

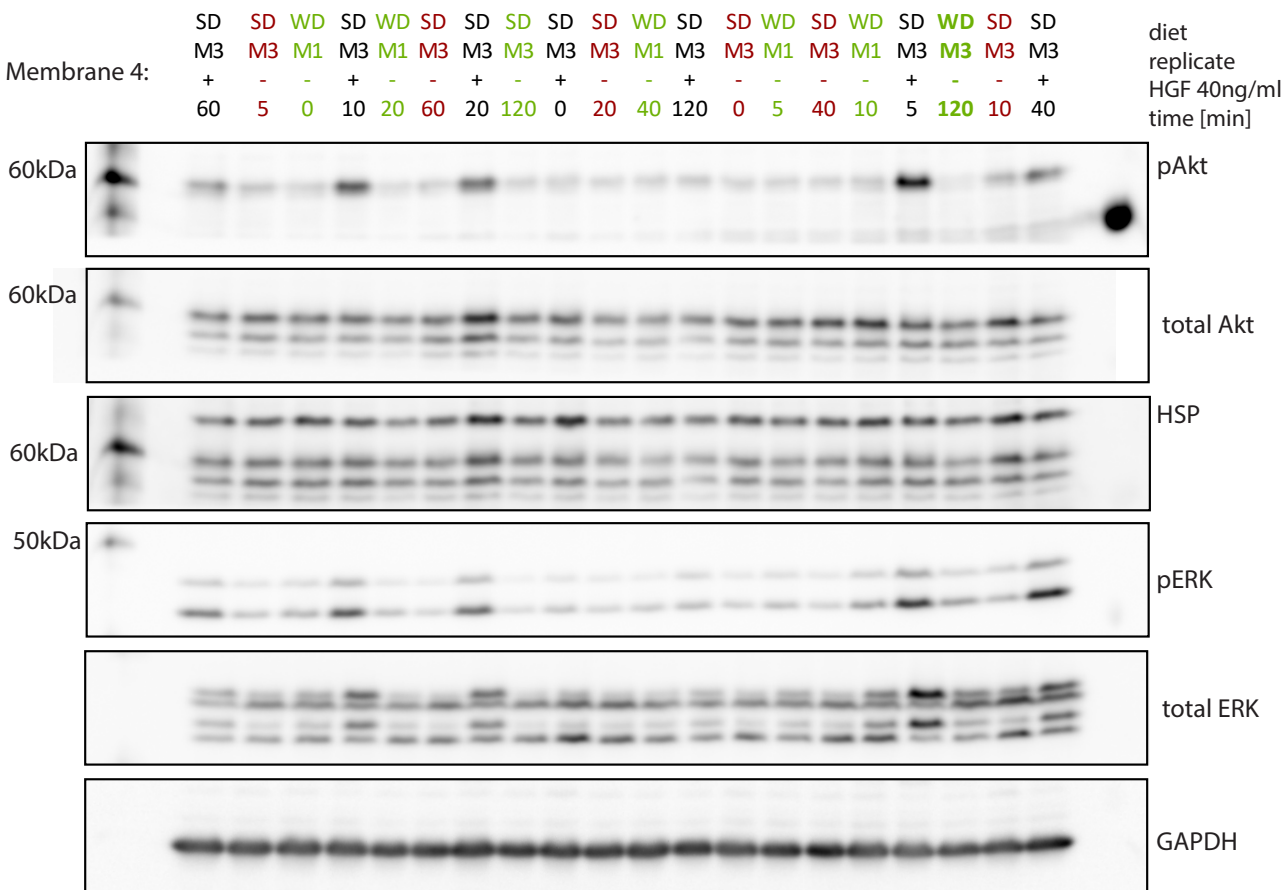

Supplement: Supplementary file 9 — Source Data Fig. 2 [file 44320_2023_7_MOESM9_ESM.zip › Figure 2/2C/Gel4_B1_pAkt_tAkt_pERK_tERK.pdf]

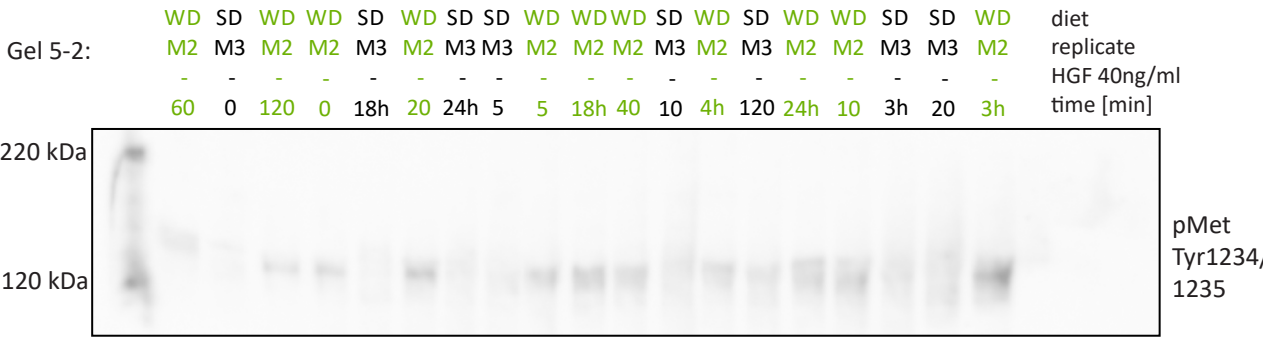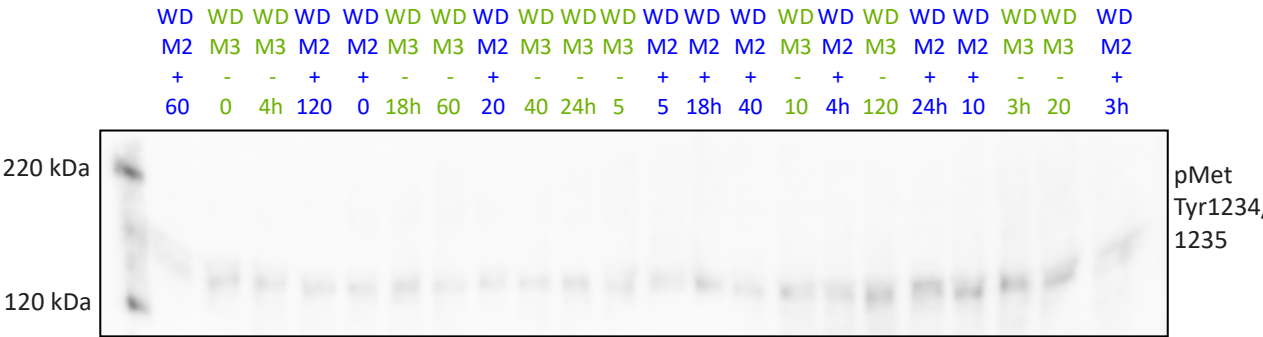

Supplement: Supplementary file 9 — Source Data Fig. 2 [file 44320_2023_7_MOESM9_ESM.zip › Figure 2/2C/Gel5-2_6-2_B2_pMet.pdf]

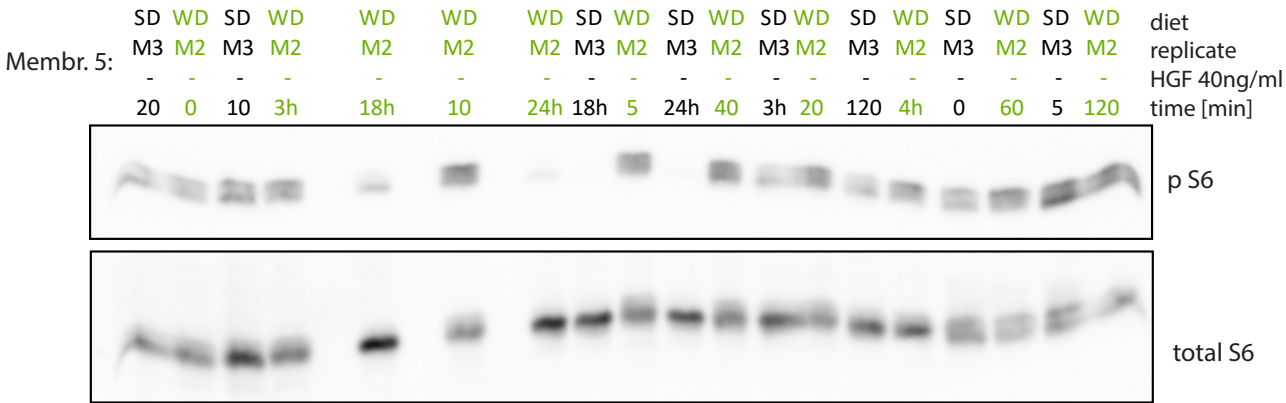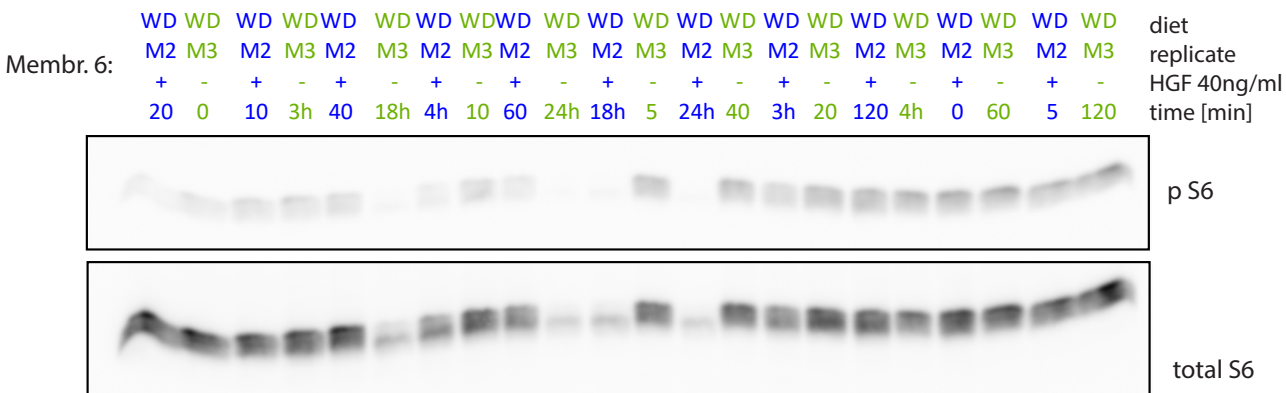

Supplement: Supplementary file 9 — Source Data Fig. 2 [file 44320_2023_7_MOESM9_ESM.zip › Figure 2/2C/Gel5_6_B2_pS6_tS6.pdf]

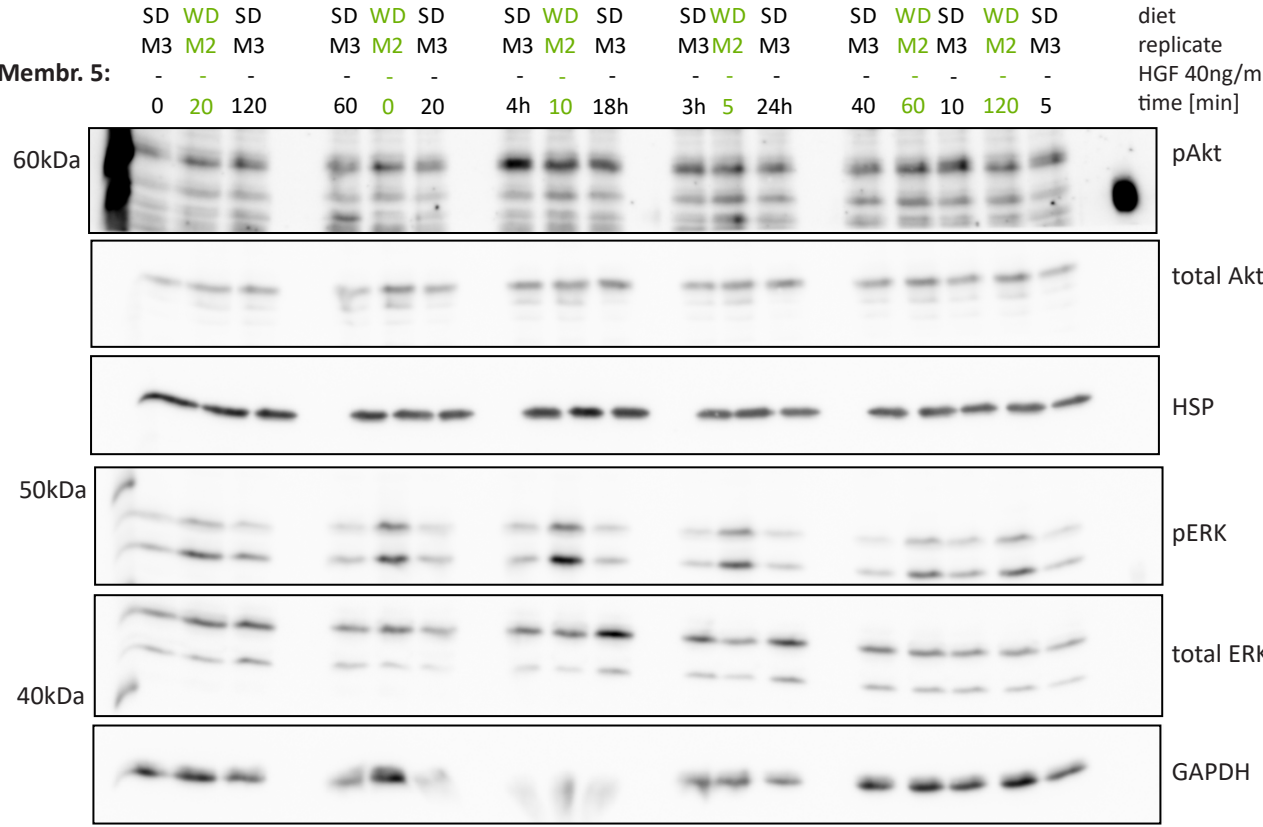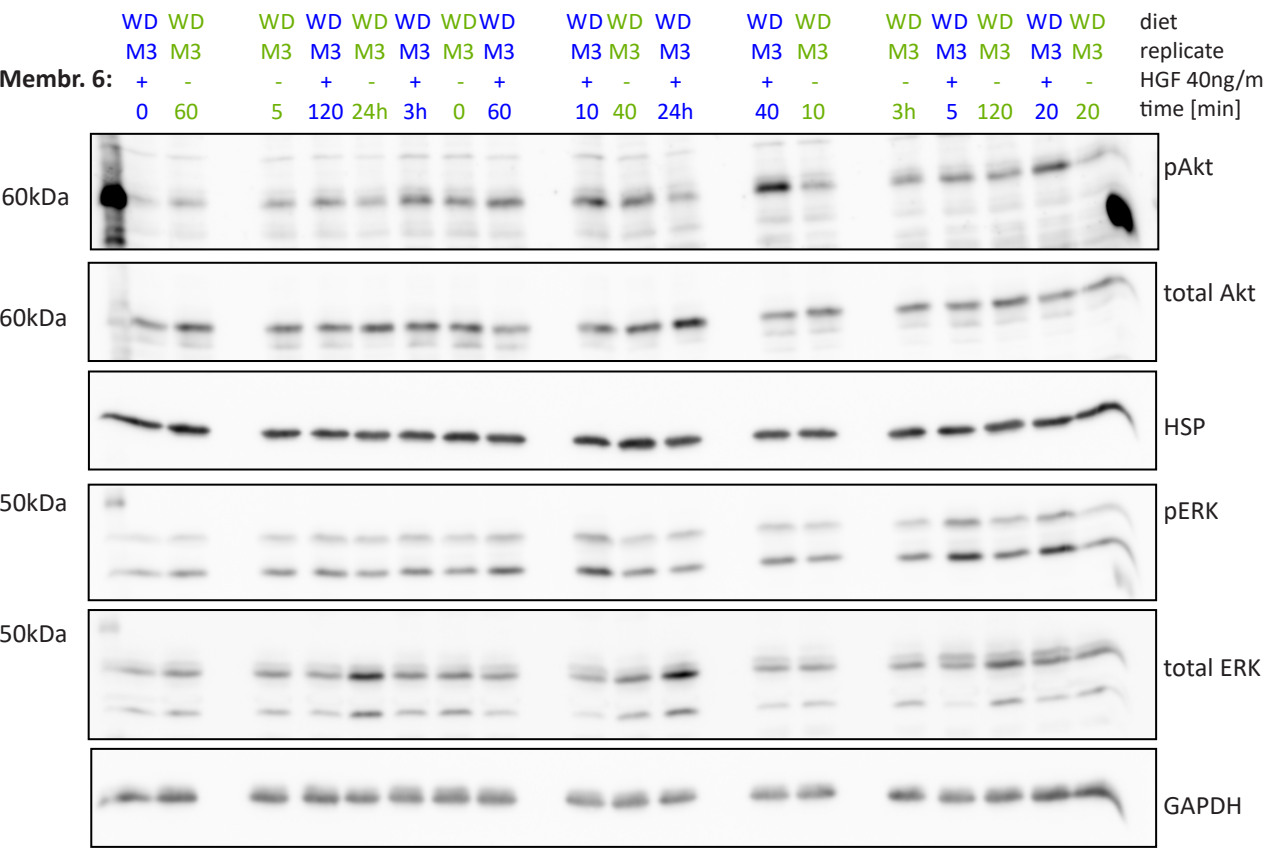

Supplement: Supplementary file 9 — Source Data Fig. 2 [file 44320_2023_7_MOESM9_ESM.zip › Figure 2/2C/Gel5_6_B3a_pAkt_tAkt_pERK_tERK.pdf]

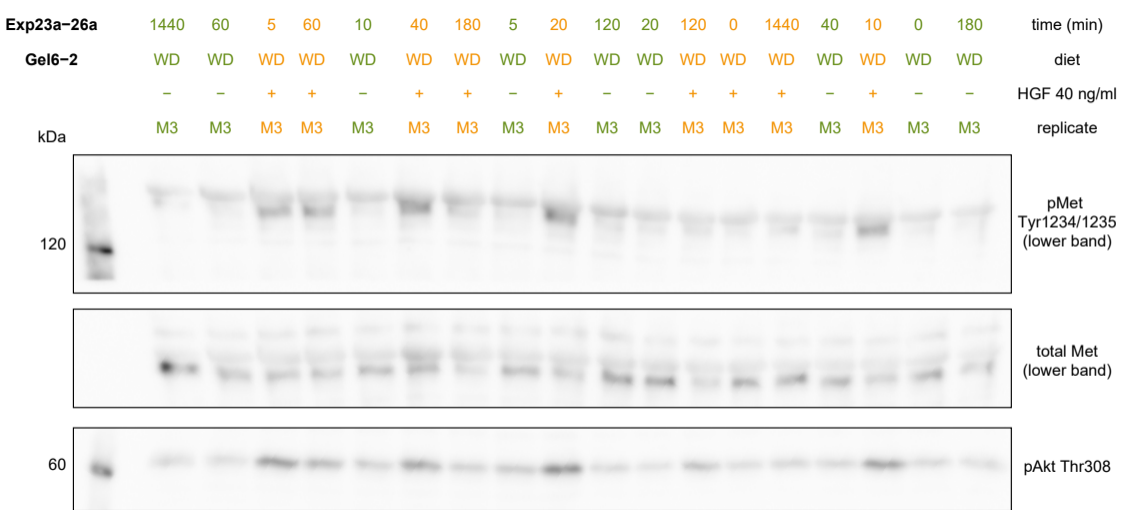

Supplement: Supplementary file 9 — Source Data Fig. 2 [file 44320_2023_7_MOESM9_ESM.zip › Figure 2/2C/Gel6-2_B3a_pMet_tMet_pAktT308.pdf]
