# Supplementary material for: Basal MET phosphorylation is an indicator of hepatocyte dysregulation in liver disease
Source: Mol Syst Biol. 2024 Jan 12;20(3):187–216. doi: 10.1038/s44320-023-00007-4 (PMC10912216; doi:10.1038/s44320-023-00007-4)
Supplement: Supplementary file 9 — Source Data Fig. 2 [file 44320_2023_7_MOESM9_ESM.zip › Figure 2/2B/Gel1-2_B3b_pMet_tMet_pAktT308.pdf]

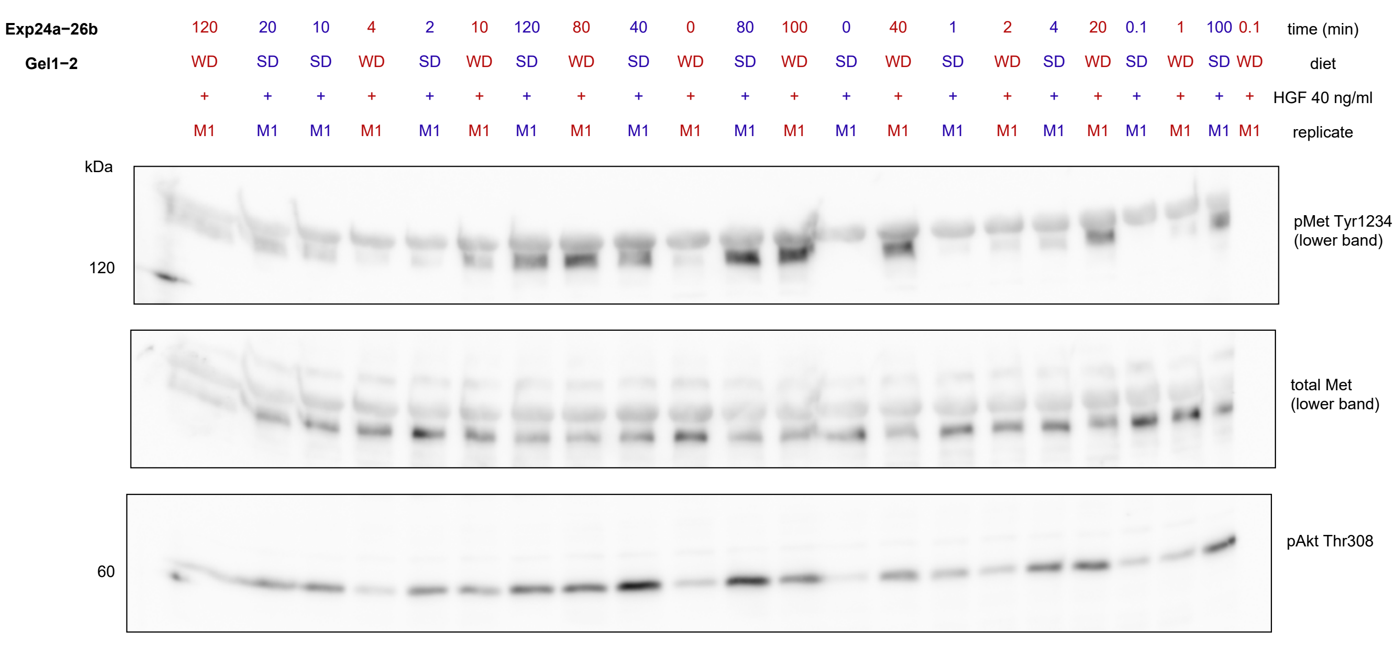
Exp24a−26b
Gel1−2
120 20 10 4 2 10 120 80 40 0 80 100 0 40 1 2 4 20 0.1 1 100 0.1 time (min)
WD SD SD WD SD WD SD WD SD WD SD WD SD WD SD WD SD WD SD WD SD WD diet
+ HGF 40 ng/ml
M1 M1 M1 M1 M1 M1 M1 M1 M1 M1 M1 M1 M1 M1 M1 M1 M1 M1 M1 M1 M1 M1 replicate
kDa
120
pMet Tyr1234 (lower band)
total Met (lower band)
60
pAkt Thr308
