# Supplementary material for: Basal MET phosphorylation is an indicator of hepatocyte dysregulation in liver disease
Source: Mol Syst Biol. 2024 Jan 12;20(3):187–216. doi: 10.1038/s44320-023-00007-4 (PMC10912216; doi:10.1038/s44320-023-00007-4)
Supplement: Supplementary file 9 — Source Data Fig. 2 [file 44320_2023_7_MOESM9_ESM.zip › Figure 2/2B/Gel1_2_B3b_pAkt_tAkt_pERK_tERK.pdf]

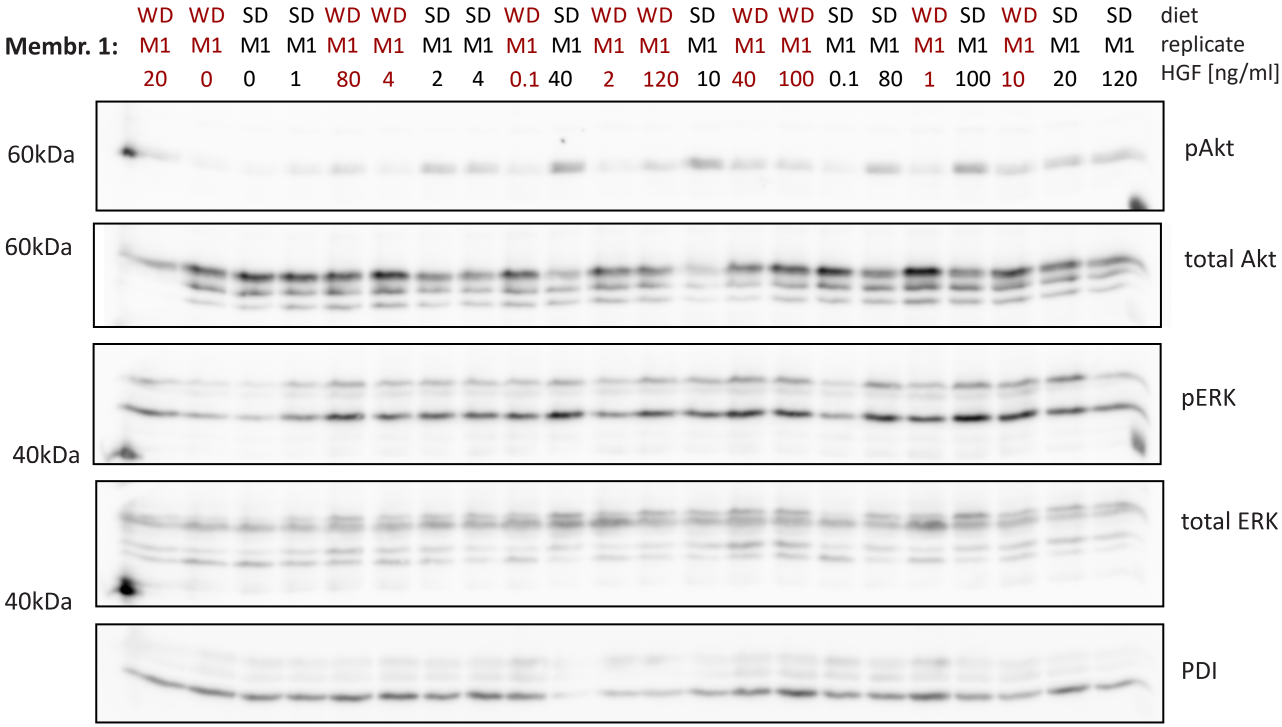
Membr. 1:
WD WD SD SD WD WD SD SD WD SD WD WD SD WD WD SD SD WD SD WD SD SD diet
M1 M1 M1 M1 M1 M1 M1 M1 M1 M1 M1 M1 M1 M1 M1 M1 M1 M1 M1 M1 M1 M1 replicate
20 0 0 1 80 4 2 4 0.1 40 2 120 10 40 100 0.1 80 1 100 10 20 120 HGF [ng/ml]
60kDa
pAkt
60kDa
total Akt
pERK
40kDa
total ERK
40kDa
PDI

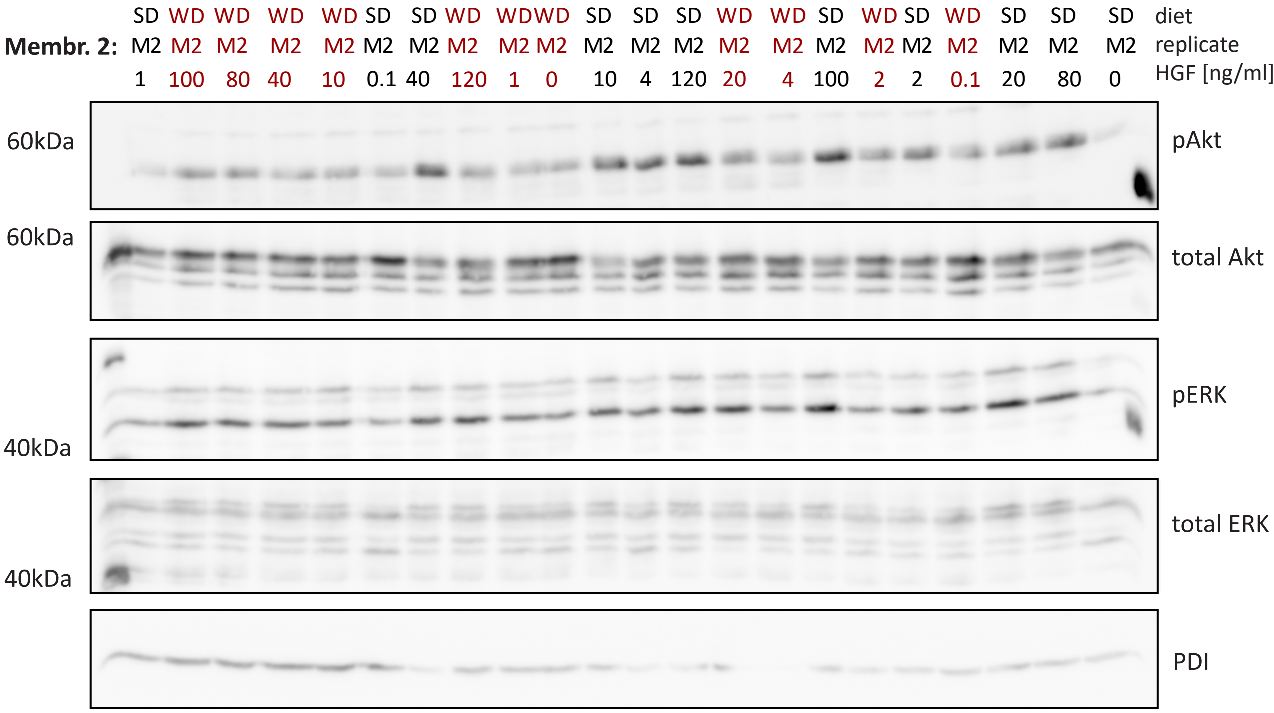
Membr. 2:
SD WD WD WD WD SD SD WD WD WD SD SD SD WD WD SD WD SD WD SD SD SD diet
M2 M2 M2 M2 M2 M2 M2 M2 M2 M2 M2 M2 M2 M2 M2 M2 M2 M2 M2 M2 M2 M2 replicate
1 100 80 40 10 0.1 40 120 1 0 10 4 120 20 4 100 2 2 0.1 20 80 0 HGF [ng/ml]
60kDa
pAkt
60kDa
total Akt
pERK
40kDa
total ERK
40kDa
PDI
