# Supplementary material for: Basal MET phosphorylation is an indicator of hepatocyte dysregulation in liver disease
Source: Mol Syst Biol. 2024 Jan 12;20(3):187–216. doi: 10.1038/s44320-023-00007-4 (PMC10912216; doi:10.1038/s44320-023-00007-4)
Supplement: Supplementary file 9 — Source Data Fig. 2 [file 44320_2023_7_MOESM9_ESM.zip › Figure 2/2B/Gel2-2_B3b_pMet_tMet_pAktT308.pdf]

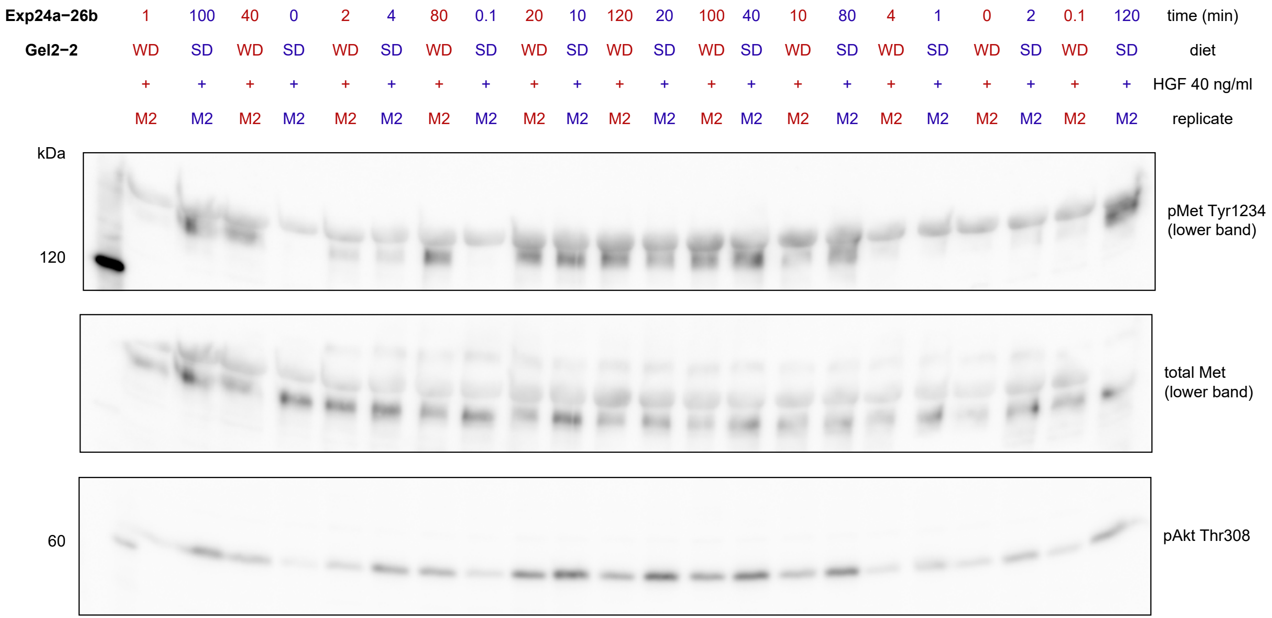
Exp24a−26b
Gel2−2
1 100 40 0 2 4 80 0.1 20 10 120 20 100 40 10 80 4 1 0 2 0.1 120 time (min)
WD SD WD SD WD SD WD SD WD SD WD SD WD SD WD SD WD SD WD SD WD SD diet
+ HGF 40 ng/ml
M2 M2 M2 M2 M2 M2 M2 M2 M2 M2 M2 M2 M2 M2 M2 M2 M2 M2 M2 M2 M2 M2 replicate
kDa
120
pMet Tyr1234 (lower band)
total Met (lower band)
60
pAkt Thr308
