# Supplementary material for: Basal MET phosphorylation is an indicator of hepatocyte dysregulation in liver disease
Source: Mol Syst Biol. 2024 Jan 12;20(3):187–216. doi: 10.1038/s44320-023-00007-4 (PMC10912216; doi:10.1038/s44320-023-00007-4)
Supplement: Supplementary file 9 — Source Data Fig. 2 [file 44320_2023_7_MOESM9_ESM.zip › Figure 2/2B/Gel3-2_B3b_pMet_tMet_pAktT308.pdf]

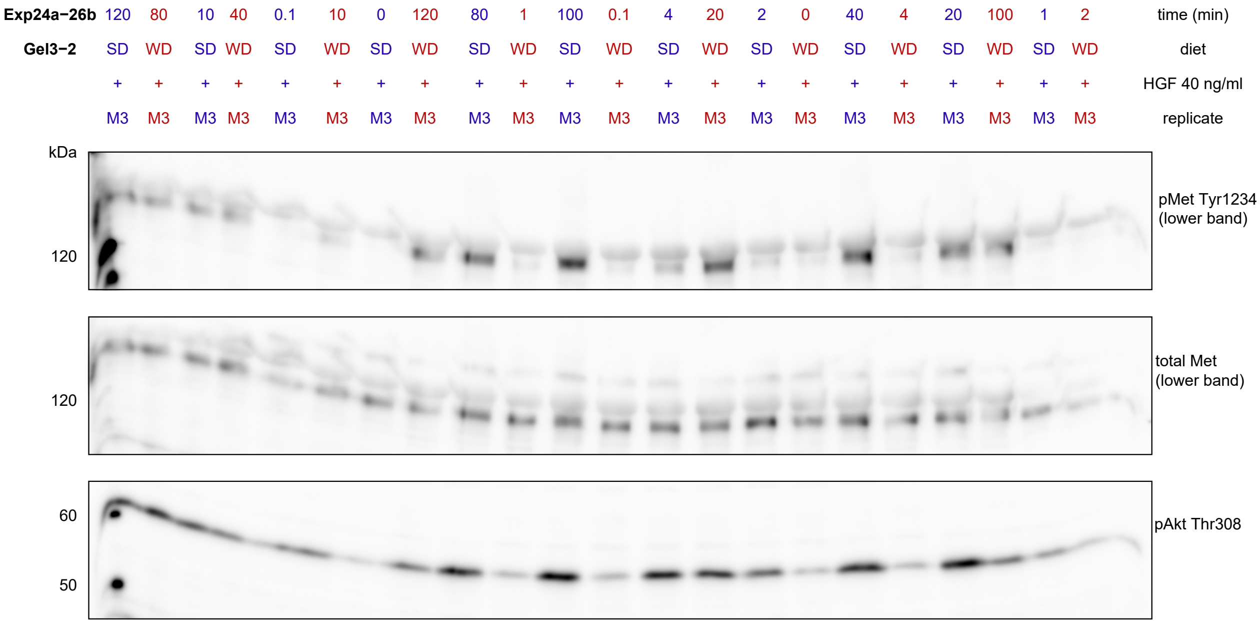
Exp24a−26b
Gel3−2
time (min)
120 80 10 40 0.1 10 0 120 80 1 100 0.1 4 20 2 0 40 4 20 100 1 2
diet
SD WD SD WD SD WD SD WD SD WD SD WD SD WD SD WD SD WD SD WD SD WD
HGF 40 ng/ml
+ +
replicate
M3 M3 M3 M3 M3 M3 M3 M3 M3 M3 M3 M3 M3 M3 M3 M3 M3 M3 M3 M3 M3 M3
kDa
120
pMet Tyr1234 (lower band)
120
total Met (lower band)
60
50
pAkt Thr308
