# Supplementary material for: Basal MET phosphorylation is an indicator of hepatocyte dysregulation in liver disease
Source: Mol Syst Biol. 2024 Jan 12;20(3):187–216. doi: 10.1038/s44320-023-00007-4 (PMC10912216; doi:10.1038/s44320-023-00007-4)
Supplement: Supplementary file 9 — Source Data Fig. 2 [file 44320_2023_7_MOESM9_ESM.zip › Figure 2/2B/Gel3_B3b_pAkt_tAkt_pERK_tERK.pdf]

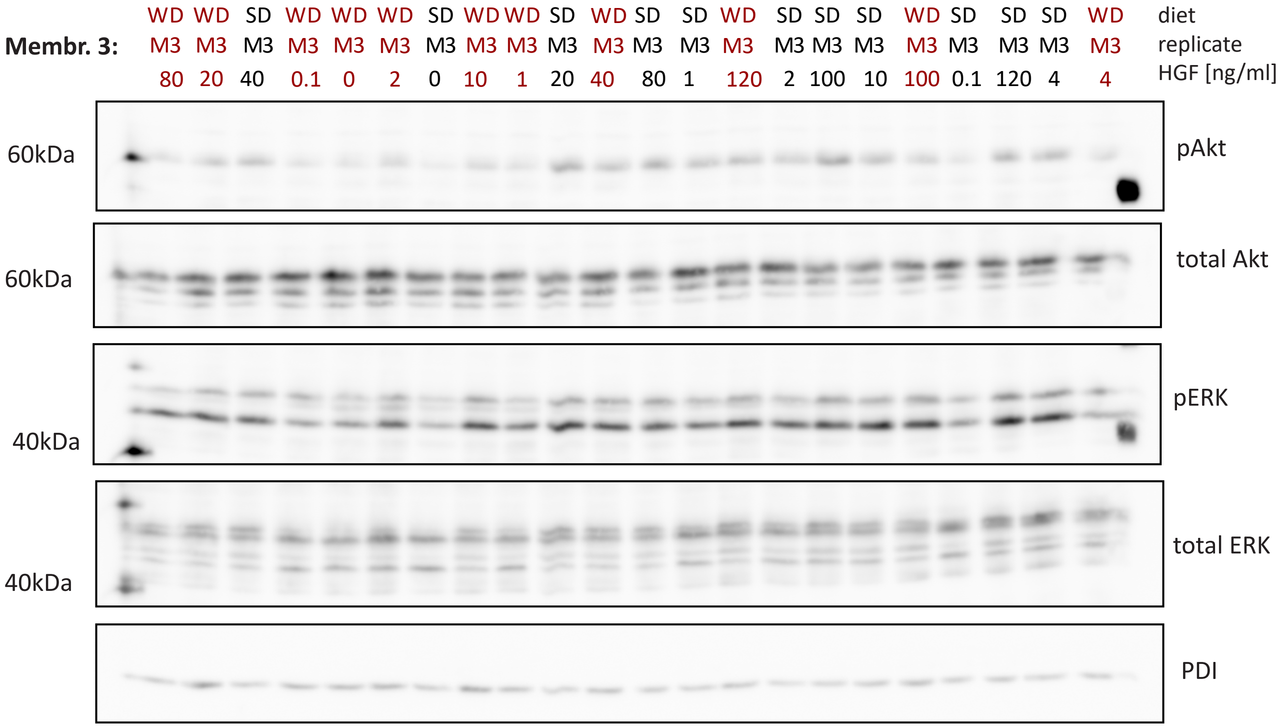
Membr. 3:
WD WD SD WD WD WD SD WD WD SD WD SD SD WD SD SD SD WD SD SD SD WD diet
M3 M3 M3 M3 M3 M3 M3 M3 M3 M3 M3 M3 M3 M3 M3 M3 M3 M3 M3 M3 M3 M3 replicate
80 20 40 0.1 0 2 0 10 1 20 40 80 1 120 2 100 10 100 0.1 120 4 4 HGF [ng/ml]
60kDa
pAkt
60kDa
total Akt
40kDa
pERK
40kDa
total ERK
PDI
