# Supplementary material for: Basal MET phosphorylation is an indicator of hepatocyte dysregulation in liver disease
Source: Mol Syst Biol. 2024 Jan 12;20(3):187–216. doi: 10.1038/s44320-023-00007-4 (PMC10912216; doi:10.1038/s44320-023-00007-4)
Supplement: Supplementary file 9 — Source Data Fig. 2 [file 44320_2023_7_MOESM9_ESM.zip › Figure 2/2C/Gel1-2_2-2_3-2_4-2_B2_pMet.pdf]

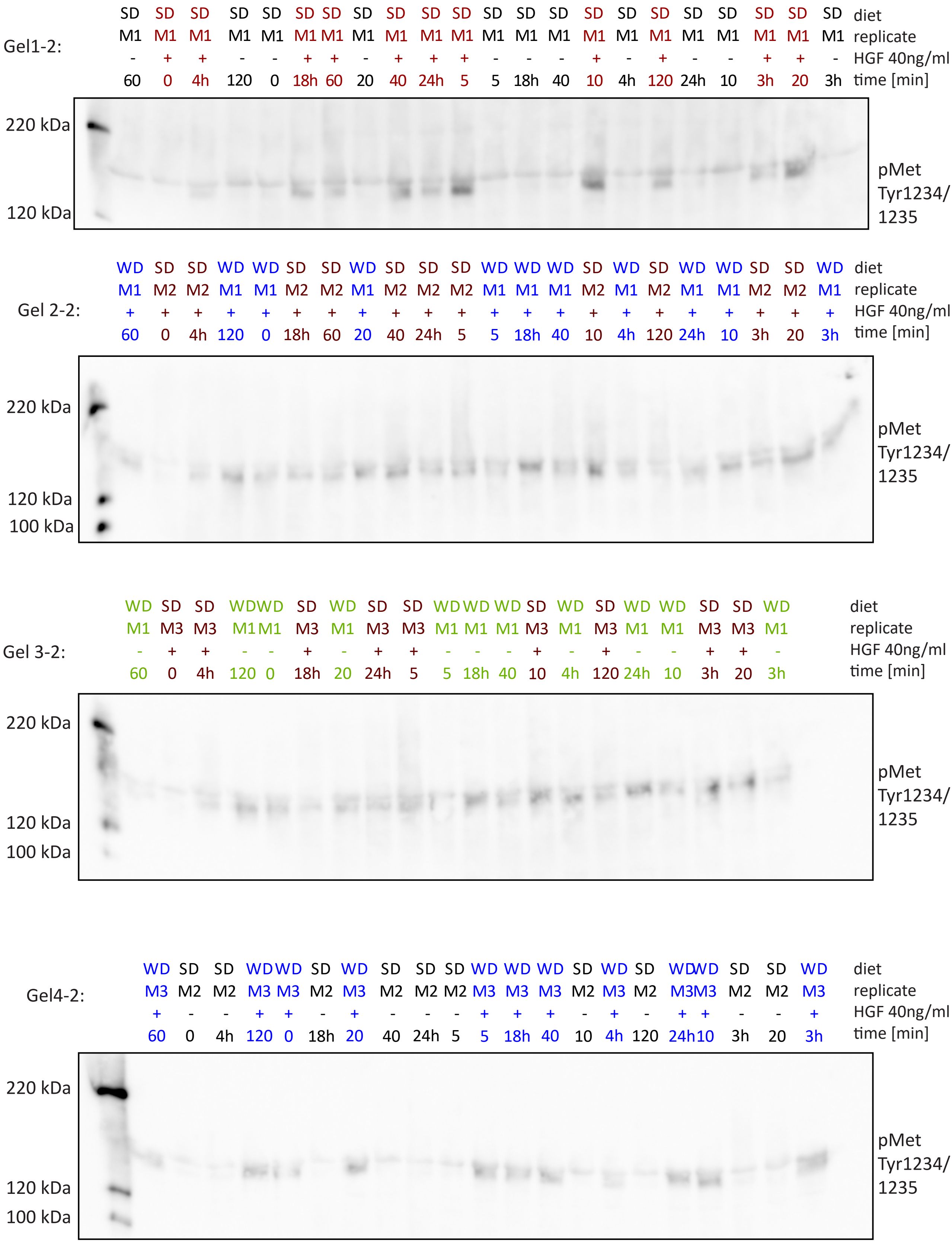
Gel1-2:
SD SD SD SD SD SD SD SD SD SD SD SD SD SD SD SD SD SD SD SD SD SD diet
M1 M1 M1 M1 M1 M1 M1 M1 M1 M1 M1 M1 M1 M1 M1 M1 M1 M1 M1 M1 M1 M1 replicate
- + + - - + + - + + + - - - + - + - - + + - HGF 40ng/ml
60 0 4h 120 0 18h 60 20 40 24h 5 5 18h 40 10 4h 120 24h 10 3h 20 3h time [min]
220 kDa
120 kDa
pMet Tyr1234/ 1235
Gel 2-2:
WD SD SD WD WD SD SD WD SD SD SD WD WD WD SD WD SD WD WD SD SD WD diet
M1 M2 M2 M1 M1 M2 M2 M1 M2 M2 M2 M1 M1 M1 M2 M1 M2 M1 M1 M2 M2 M1 replicate
+ HGF 40ng/ml
60 0 4h 120 0 18h 60 20 40 24h 5 5 18h 40 10 4h 120 24h 10 3h 20 3h time [min]
220 kDa
120 kDa
100 kDa
pMet Tyr1234/ 1235
Gel 3-2:
WD SD SD WDWD SD WD SD SD WDWD WD SD WD SD WD WD SD SD WD diet
M1 M3 M3 M1 M1 M3 M1 M3 M3 M1 M1 M1 M3 M1 M3 M1 M1 M3 M3 M1 replicate
- + + - - + - + + - - - + - + - - + + - HGF 40ng/ml
60 0 4h 120 0 18h 20 24h 5 5 18h 40 10 4h 120 24h 10 3h 20 3h time [min]
220 kDa
120 kDa
100 kDa
pMet Tyr1234/ 1235
Gel4-2:
WD SD SD WDWD SD WD SD SD SD WD WD WD SD WD SD WDWD SD SD WD diet
M3 M2 M2 M3 M3 M2 M3 M2 M2 M2 M3 M3 M3 M2 M3 M2 M3M3 M2 M2 M3 replicate
+ - - + + - + - - - + + + - + - + + - - + HGF 40ng/ml
60 0 4h 120 0 18h 20 40 24h 5 5 18h 40 10 4h 120 24h10 3h 20 3h time [min]
220 kDa
120 kDa
100 kDa
pMet Tyr1234/ 1235
