# Supplementary material for: Basal MET phosphorylation is an indicator of hepatocyte dysregulation in liver disease
Source: Mol Syst Biol. 2024 Jan 12;20(3):187–216. doi: 10.1038/s44320-023-00007-4 (PMC10912216; doi:10.1038/s44320-023-00007-4)
Supplement: Supplementary file 9 — Source Data Fig. 2 [file 44320_2023_7_MOESM9_ESM.zip › Figure 2/2C/Gel1-2_2-2_3-2_B2_tMet_pAktT308.pdf]

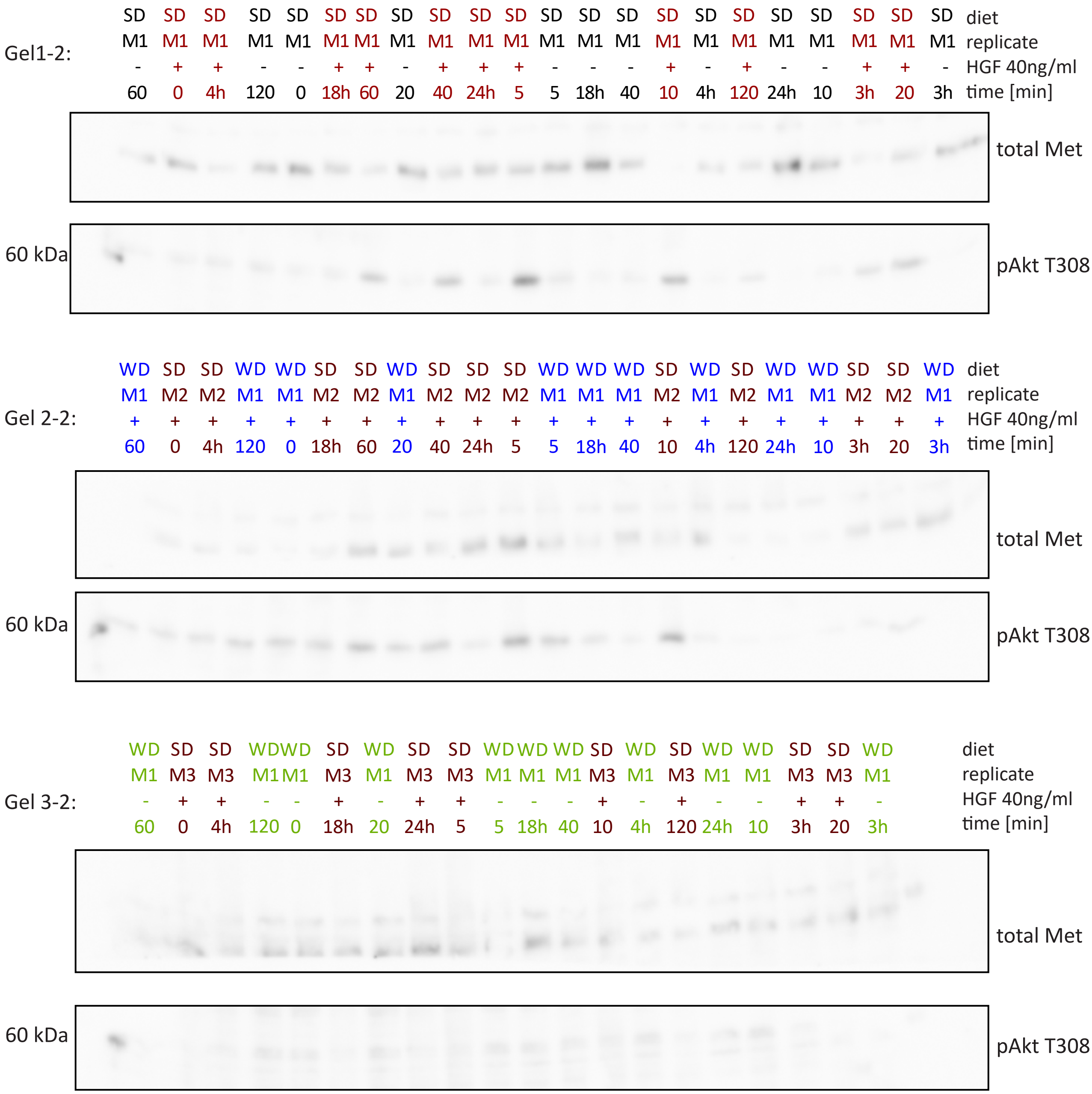
Gel1-2:
SD SD SD SD SD SD SD SD SD SD SD SD SD SD SD SD SD SD SD SD SD SD diet
M1 M1 M1 M1 M1 M1 M1 M1 M1 M1 M1 M1 M1 M1 M1 M1 M1 M1 M1 M1 M1 M1 replicate
- + + - - + + - + + + - - - + - + - - + + - HGF 40ng/ml
60 0 4h 120 0 18h 60 20 40 24h 5 5 18h 40 10 4h 120 24h 10 3h 20 3h time [min]
total Met
60 kDa
pAkt T308
Gel 2-2:
WD SD SD WD WD SD SD WD SD SD SD WD WD WD SD WD SD WD WD SD SD WD diet
M1 M2 M2 M1 M1 M2 M2 M1 M2 M2 M2 M1 M1 M1 M2 M1 M2 M1 M1 M2 M2 M1 replicate
+ + + + + + + + + + + + + + + + + + + + + + HGF 40ng/ml
60 0 4h 120 0 18h 60 20 40 24h 5 5 18h 40 10 4h 120 24h 10 3h 20 3h time [min]
total Met
60 kDa
pAkt T308
Gel 3-2:
WD SD SD WD WD SD WD SD SD WD WD WD SD WD SD WD WD SD SD WD diet
M1 M3 M3 M1 M1 M3 M1 M3 M3 M1 M1 M1 M3 M1 M3 M1 M1 M3 M3 M1 replicate
- + + - - + - + + - - - + - + - - + + - HGF 40ng/ml
60 0 4h 120 0 18h 20 24h 5 5 18h 40 10 4h 120 24h 10 3h 20 3h time [min]
total Met
60 kDa
pAkt T308
