# Supplementary material for: Basal MET phosphorylation is an indicator of hepatocyte dysregulation in liver disease
Source: Mol Syst Biol. 2024 Jan 12;20(3):187–216. doi: 10.1038/s44320-023-00007-4 (PMC10912216; doi:10.1038/s44320-023-00007-4)
Supplement: Supplementary file 9 — Source Data Fig. 2 [file 44320_2023_7_MOESM9_ESM.zip › Figure 2/2C/Gel1-2_B1_pAktT308.pdf]

Exp18a-19a Gel1-2

10	5	20	40	120	40	0	10	60	5	0	60	120	40	20	5	60	10	0	120	20	time (min)
SD	WD	SD	SD	SD	WD	SD	SD	WD	SD	WD	SD	SD	SD	WD	SD	SD	WD	SD	WD	SD	diet
–	+	+	–	+	+	–	+	+	–	+	+	–	+	+	+	–	+	+	+	–	condition
M1	M1	M1	M1	M1	M1	M1	M1	M1	M1	M1	M1	M1	M1	M1	M1	M1	M1	M1	M1	M1	replicate

kDa

60

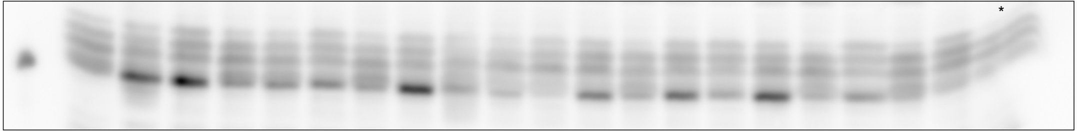

* spilled in 2 lanes

pAkt Thr308 (lowest band)
