# Supplementary material for: Basal MET phosphorylation is an indicator of hepatocyte dysregulation in liver disease
Source: Mol Syst Biol. 2024 Jan 12;20(3):187–216. doi: 10.1038/s44320-023-00007-4 (PMC10912216; doi:10.1038/s44320-023-00007-4)
Supplement: Supplementary file 9 — Source Data Fig. 2 [file 44320_2023_7_MOESM9_ESM.zip › Figure 2/2C/Gel1-2_B3a_pMet_tMet_pAktT308.pdf]

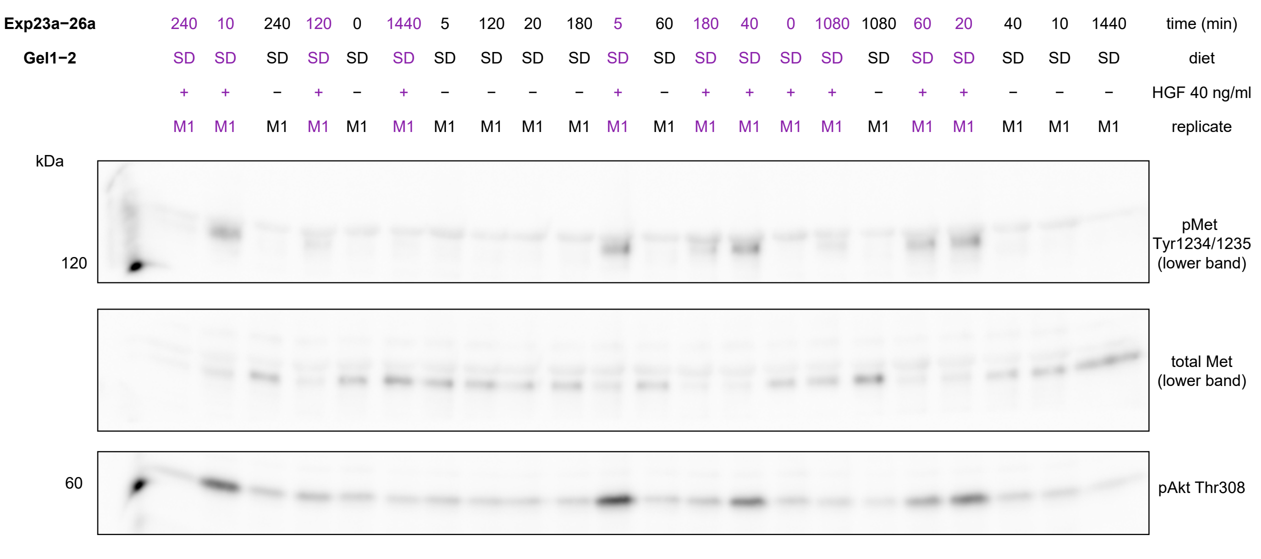
Exp23a−26a
Gel1−2
240 10 240 120 0 1440 5 120 20 180 5 60 180 40 0 1080 1080 60 20 40 10 1440
time (min)
SD SD SD SD SD SD SD SD SD SD SD SD SD SD SD SD SD SD SD SD SD SD
diet
+ + − + − + − − − − + − + + + + − + + − − −
HGF 40 ng/ml
M1 M1 M1 M1 M1 M1 M1 M1 M1 M1 M1 M1 M1 M1 M1 M1 M1 M1 M1 M1 M1 M1
replicate
kDa
120
60
pMet Tyr1234/1235 (lower band)
total Met (lower band)
pAkt Thr308
