# Supplementary material for: Basal MET phosphorylation is an indicator of hepatocyte dysregulation in liver disease
Source: Mol Syst Biol. 2024 Jan 12;20(3):187–216. doi: 10.1038/s44320-023-00007-4 (PMC10912216; doi:10.1038/s44320-023-00007-4)
Supplement: Supplementary file 9 — Source Data Fig. 2 [file 44320_2023_7_MOESM9_ESM.zip › Figure 2/2C/Gel1_2_3_4_B2_pS6_tS6.pdf]

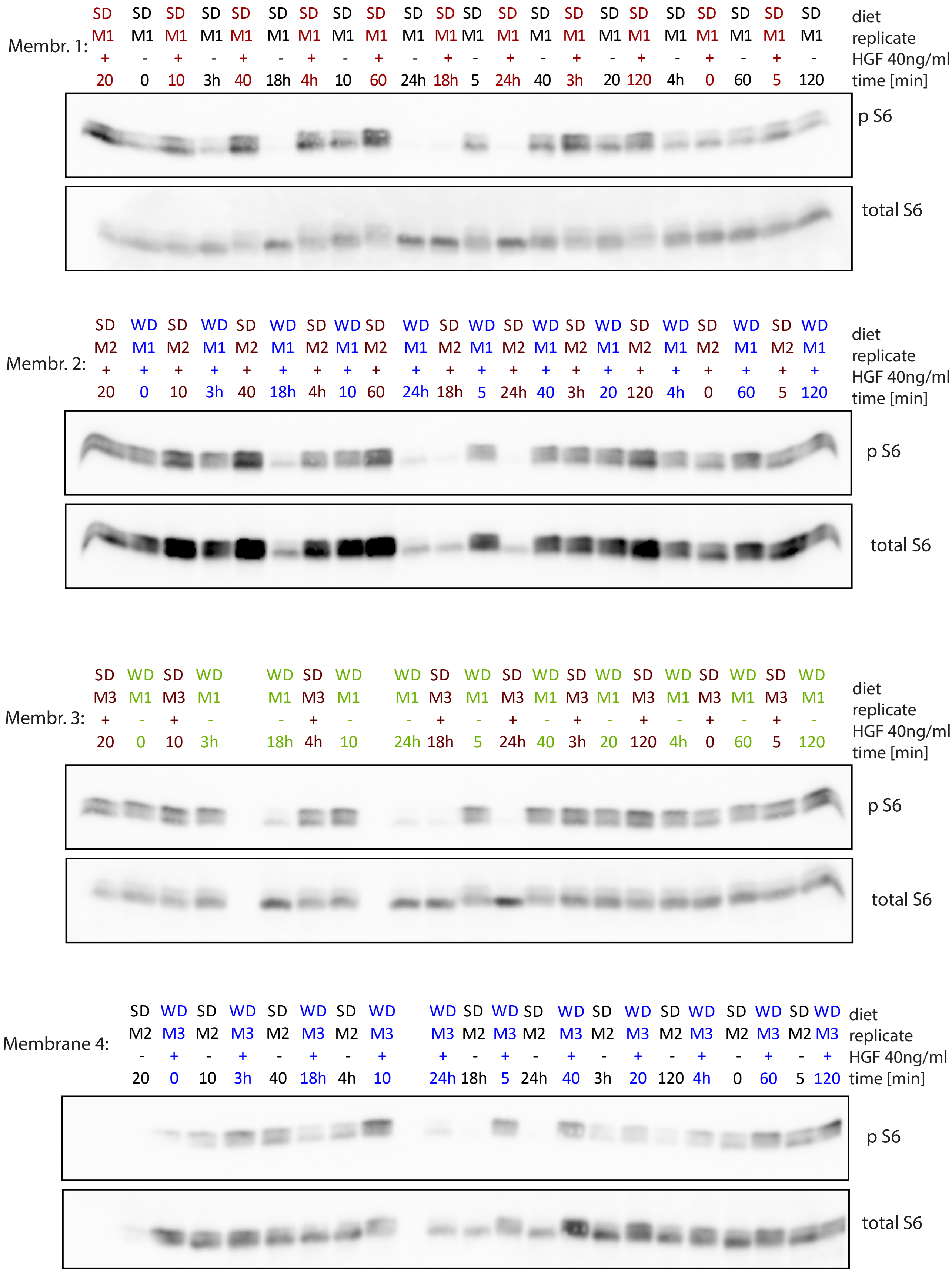
Membr. 1:
SD SD SD SD SD SD SD SD SD SD SD SD SD SD SD SD SD SD SD SD SD SD
M1 M1 M1 M1 M1 M1 M1 M1 M1 M1 M1 M1 M1 M1 M1 M1 M1 M1 M1 M1 M1 M1
+ - + - + - + - + - + - + - + - + - + - + -
20 0 10 3h 40 18h 4h 10 60 24h 18h 5 24h 40 3h 20 120 4h 0 60 5 120
diet
replicate
HGF 40ng/ml
time [min]
p S6
total S6
Membr. 2:
SD WD SD WD SD WD SD WD SD WD SD WD SD WD SD WD SD WD SD WD SD WD
M2 M1 M2 M1 M2 M1 M2 M1 M2 M1 M2 M1 M2 M1 M2 M1 M2 M1 M2 M1 M2 M1
+ +
20 0 10 3h 40 18h 4h 10 60 24h 18h 5 24h 40 3h 20 120 4h 0 60 5 120
diet
replicate
HGF 40ng/ml
time [min]
p S6
total S6
Membr. 3:
SD WD SD WD WD SD WD WD SD WD SD WD SD WD SD WD SD WD SD WD
M3 M1 M3 M1 M1 M3 M1 M1 M3 M1 M3 M1 M3 M1 M3 M1 M3 M1 M3 M1
+ - + - - + - - + - + - + - + - + - + -
20 0 10 3h 18h 4h 10 24h 18h 5 24h 40 3h 20 120 4h 0 60 5 120
diet
replicate
HGF 40ng/ml
time [min]
p S6
total S6
Membrane 4:
SD WD SD WD SD WD SD WD WD SD WD SD WD SD WD SD WD SD WD SD WD
M2 M3 M2 M3 M2 M3 M2 M3 M3 M2 M3 M2 M3 M2 M3 M2 M3 M2 M3 M2 M3
- + - + - + - + + - + - + - + - + - + - +
20 0 10 3h 40 18h 4h 10 24h 18h 5 24h 40 3h 20 120 4h 0 60 5 120
diet
replicate
HGF 40ng/ml
time [min]
p S6
total S6
