# Supplementary material for: Basal MET phosphorylation is an indicator of hepatocyte dysregulation in liver disease
Source: Mol Syst Biol. 2024 Jan 12;20(3):187–216. doi: 10.1038/s44320-023-00007-4 (PMC10912216; doi:10.1038/s44320-023-00007-4)
Supplement: Supplementary file 9 — Source Data Fig. 2 [file 44320_2023_7_MOESM9_ESM.zip › Figure 2/2C/Gel1_2_B3a_pAkt_tAkt_pERK_tERK.pdf]

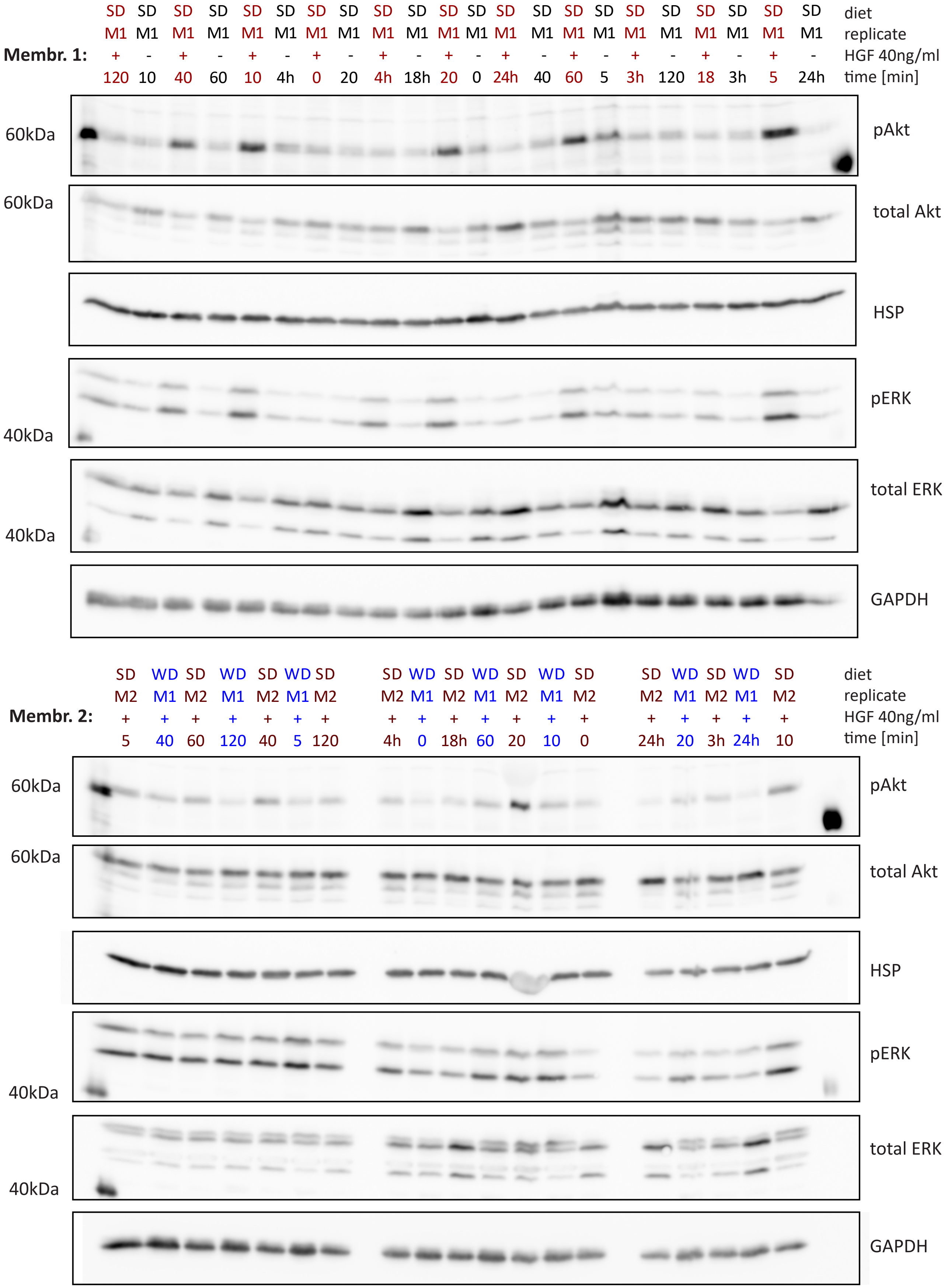
SD SD SD SD SD SD SD SD SD SD SD SD SD SD SD SD SD SD SD SD SD SD
M1 M1 M1 M1 M1 M1 M1 M1 M1 M1 M1 M1 M1 M1 M1 M1 M1 M1 M1 M1 M1 M1
Membr. 1:
+ - + - + - + - + - + - + - + - + - + - + -
120 10 40 60 10 4h 0 20 4h 18h 20 0 24h 40 60 5 3h 120 18 3h 5 24h
diet
replicate
HGF 40ng/ml
time [min]
60kDa
pAkt
60kDa
total Akt
HSP
pERK
40kDa
total ERK
40kDa
GAPDH
SD WD SD WD SD WD SD SD WD SD WD SD WD SD SD WD SD WD SD
M2 M1 M2 M1 M2 M1 M2 M2 M1 M2 M1 M2 M1 M2 M2 M1 M2 M1 M2
Membr. 2:
+ + + + + + + + + + + + + + + + + + +
5 40 60 120 40 5 120 4h 0 18h 60 20 10 0 24h 20 3h 24h 10
diet
replicate
HGF 40ng/ml
time [min]
60kDa
pAkt
60kDa
total Akt
HSP
pERK
40kDa
total ERK
40kDa
GAPDH
