# Supplementary material for: Basal MET phosphorylation is an indicator of hepatocyte dysregulation in liver disease
Source: Mol Syst Biol. 2024 Jan 12;20(3):187–216. doi: 10.1038/s44320-023-00007-4 (PMC10912216; doi:10.1038/s44320-023-00007-4)
Supplement: Supplementary file 9 — Source Data Fig. 2 [file 44320_2023_7_MOESM9_ESM.zip › Figure 2/2C/Gel1_B1_pS6_tS6.pdf]

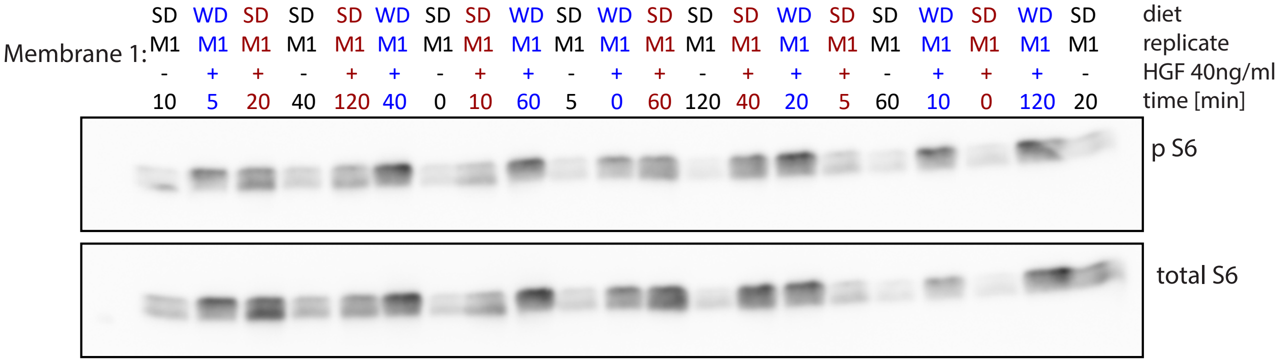
Membrane 1:
SD WD SD SD SD WD SD SD WD SD WD SD SD SD WD SD SD WD SD WD SD
M1 M1 M1 M1 M1 M1 M1 M1 M1 M1 M1 M1 M1 M1 M1 M1 M1 M1 M1 M1 M1
- + + - + + - + + - + + - + + + - + + + -
10 5 20 40 120 40 0 10 60 5 0 60 120 40 20 5 60 10 0 120 20
diet
replicate
HGF 40ng/ml
time [min]
p S6
total S6
