# Supplementary material for: Basal MET phosphorylation is an indicator of hepatocyte dysregulation in liver disease
Source: Mol Syst Biol. 2024 Jan 12;20(3):187–216. doi: 10.1038/s44320-023-00007-4 (PMC10912216; doi:10.1038/s44320-023-00007-4)

Membr. 1, batch 3:

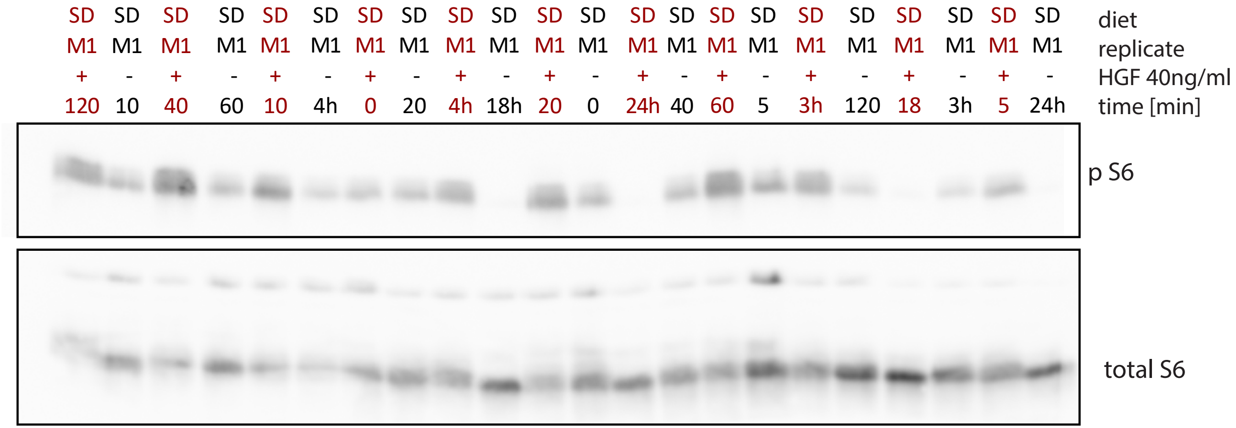

Supplement: Supplementary file 9 — Source Data Fig. 2 [file 44320_2023_7_MOESM9_ESM.zip › Figure 2/2C/Gel1_B3a_pS6_tS6.pdf]
