# Supplementary material for: Basal MET phosphorylation is an indicator of hepatocyte dysregulation in liver disease
Source: Mol Syst Biol. 2024 Jan 12;20(3):187–216. doi: 10.1038/s44320-023-00007-4 (PMC10912216; doi:10.1038/s44320-023-00007-4)
Supplement: Supplementary file 9 — Source Data Fig. 2 [file 44320_2023_7_MOESM9_ESM.zip › Figure 2/2C/Gel2-2_B1_pAktT308.pdf]

Exp18a-19a
Gel2-2

20	0	10	40	120	0	20	5	60	10	40	120	20	10	5	60	5	60	40	0	120	time (min)
WD	SD	WD	SD	WD	WD	SD	WD	WD	SD	WD	SD	WD	WD	SD	WD	WD	SD	WD	WD	WD	diet
+	–	–	–	+	–	–	+	–	–	+	–	–	+	–	+	–	–	–	+	–	condition
M2	M2	M2	M2	M2	M2	M2	M2	M2	M2	M2	M2	M2	M2	M2	M2	M2	M2	M2	M2	M2	replicate

kDa

60

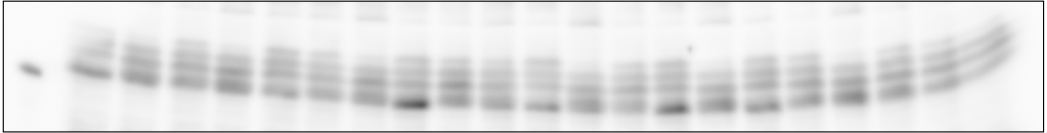

pAkt Thr308
(lowest band)
