# Supplementary material for: Basal MET phosphorylation is an indicator of hepatocyte dysregulation in liver disease
Source: Mol Syst Biol. 2024 Jan 12;20(3):187–216. doi: 10.1038/s44320-023-00007-4 (PMC10912216; doi:10.1038/s44320-023-00007-4)
Supplement: Supplementary file 9 — Source Data Fig. 2 [file 44320_2023_7_MOESM9_ESM.zip › Figure 2/2C/Gel2_B1_pS6_tS6.pdf]

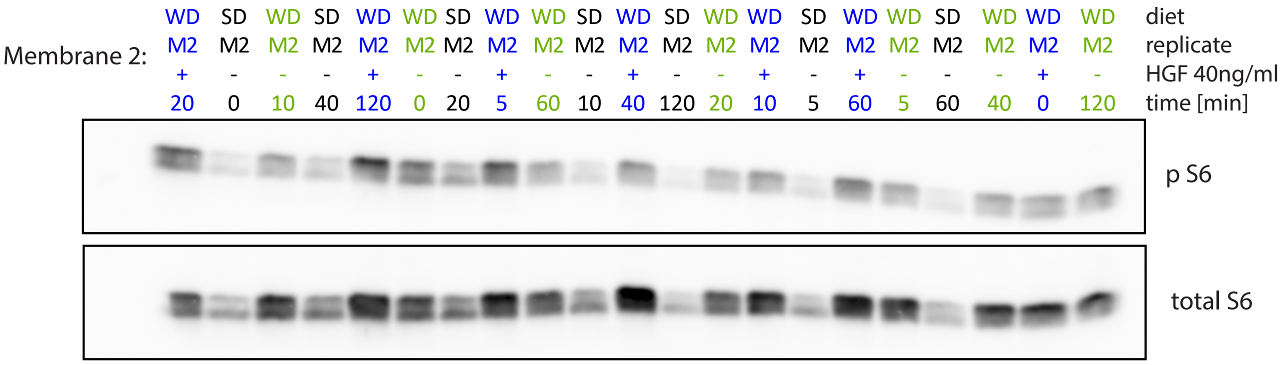
Membrane 2:
WD SD WD SD WD WD SD WD WD SD WD SD WD WD SD WD WD SD WD WD WD diet
M2 M2 M2 M2 M2 M2 M2 M2 M2 M2 M2 M2 M2 M2 M2 M2 M2 M2 M2 M2 M2 replicate
+ - - - + - - + - - + - - + - + - - - + - HGF 40ng/ml
20 0 10 40 120 0 20 5 60 10 40 120 20 10 5 60 5 60 40 0 120 time [min]
p S6
total S6
