# Supplementary material for: Basal MET phosphorylation is an indicator of hepatocyte dysregulation in liver disease
Source: Mol Syst Biol. 2024 Jan 12;20(3):187–216. doi: 10.1038/s44320-023-00007-4 (PMC10912216; doi:10.1038/s44320-023-00007-4)
Supplement: Supplementary file 9 — Source Data Fig. 2 [file 44320_2023_7_MOESM9_ESM.zip › Figure 2/2C/Gel2_B3a_pS6_tS6.pdf]

Membrane 2:

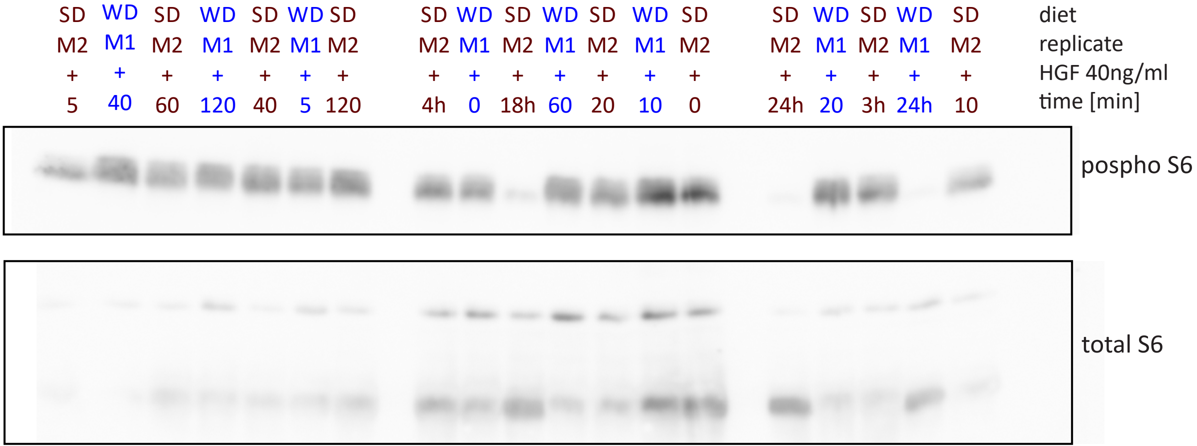
SD WD SD WD SD WD SD SD WD SD WD SD WD SD SD WD SD WD SD
M2 M1 M2 M1 M2 M1 M2 M2 M1 M2 M1 M2 M1 M2 M2 M1 M2 M1 M2
+ + + + + + + + + + + + + + + + + + +
5 40 60 120 40 5 120 4h 0 18h 60 20 10 0 24h 20 3h 24h 10
diet
replicate
HGF 40ng/ml
time [min]
pospho S6
total S6
