# Supplementary material for: Basal MET phosphorylation is an indicator of hepatocyte dysregulation in liver disease
Source: Mol Syst Biol. 2024 Jan 12;20(3):187–216. doi: 10.1038/s44320-023-00007-4 (PMC10912216; doi:10.1038/s44320-023-00007-4)
Supplement: Supplementary file 9 — Source Data Fig. 2 [file 44320_2023_7_MOESM9_ESM.zip › Figure 2/2C/Gel3_4_B3a_pAkt_tAkt_pERK_tERK.pdf]

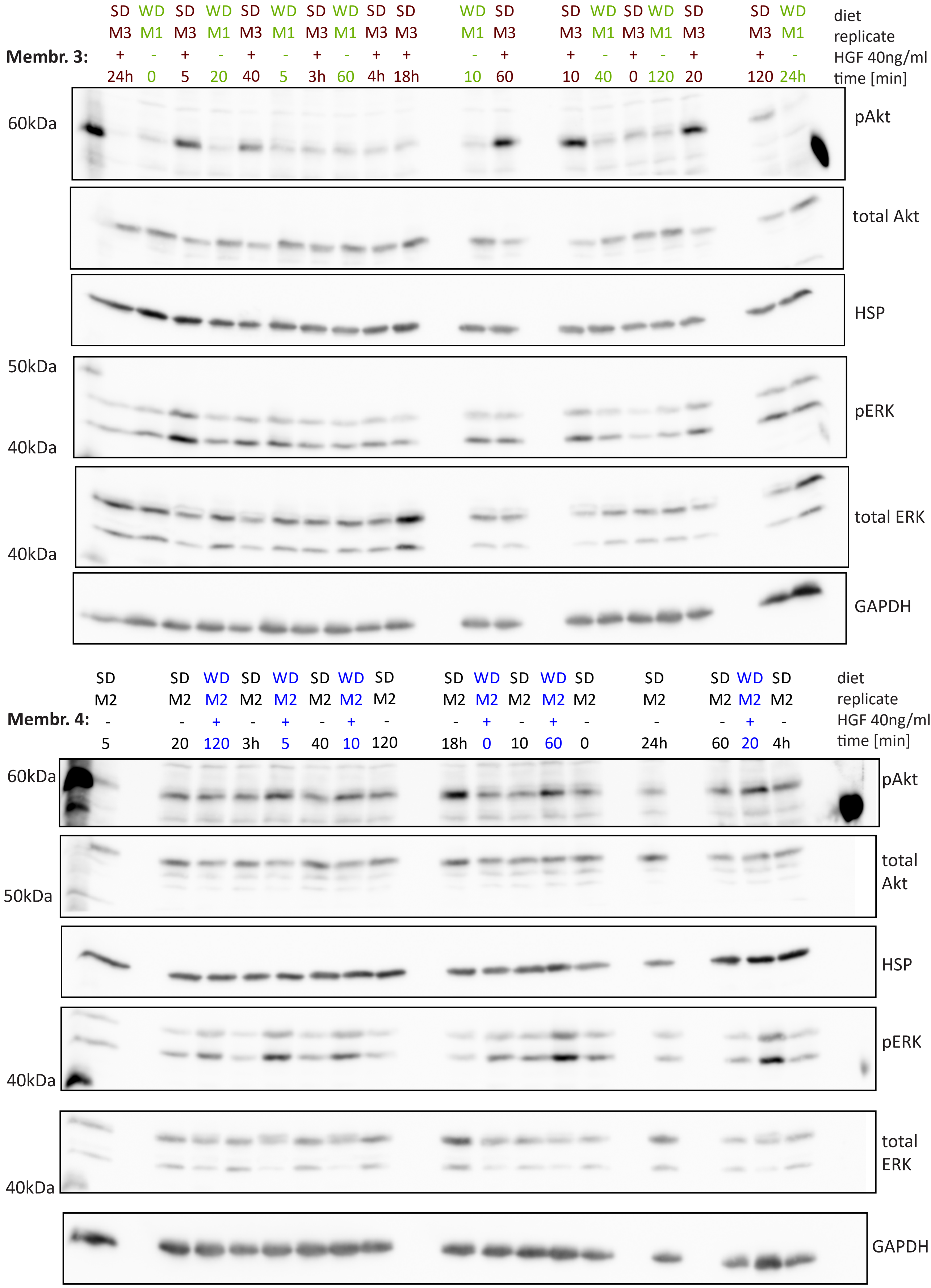

SD WD SD WD SD WD SD WD SD SD WD SD SD WD SD WD SD SD WD
M3 M1 M3 M1 M3 M1 M3 M1 M3 M3 M1 M3 M3 M1 M3 M1 M3 M3 M1
Membr. 3:
+ - + - + - + - + + - + + - + - + + -
24h 0 5 20 40 5 3h 60 4h 18h 10 60 10 40 0 120 20 120 24h
diet
replicate
HGF 40ng/ml
time [min]
60kDa
pAkt
total Akt
HSP
50kDa
40kDa
pERK
total ERK
40kDa
GAPDH
SD SD WD SD WD SD WD SD SD WD SD WD SD SD SD WD SD
M2 M2 M2 M2 M2 M2 M2 M2 M2 M2 M2 M2 M2 M2 M2 M2 M2
Membr. 4:
- - + - + - + - - + - + - - - + -
5 20 120 3h 5 40 10 120 18h 0 10 60 0 24h 60 20 4h
diet
replicate
HGF 40ng/ml
time [min]
60kDa
pAkt
total Akt
50kDa
HSP
pERK
40kDa
total ERK
40kDa
GAPDH
