# Supplementary material for: Basal MET phosphorylation is an indicator of hepatocyte dysregulation in liver disease
Source: Mol Syst Biol. 2024 Jan 12;20(3):187–216. doi: 10.1038/s44320-023-00007-4 (PMC10912216; doi:10.1038/s44320-023-00007-4)
Supplement: Supplementary file 9 — Source Data Fig. 2 [file 44320_2023_7_MOESM9_ESM.zip › Figure 2/2C/Gel3_B1_pS6_tS6.pdf]

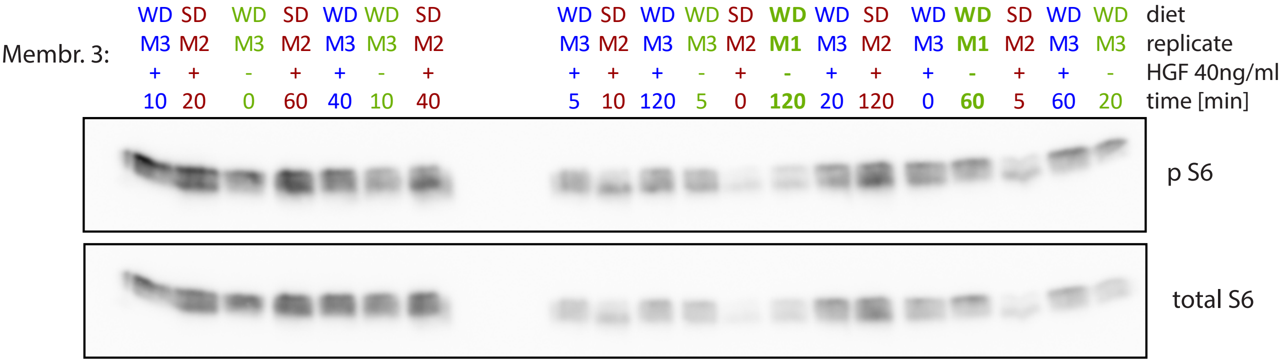
Membr. 3:
WD SD WD SD WD WD SD WD SD WD WD SD WD WD SD WD WD SD WD WD diet
M3 M2 M3 M2 M3 M3 M2 M3 M2 M3 M3 M2 M1 M3 M2 M3 M1 M2 M3 M3 replicate
+ + - + + - + + + + - + - + + + - + + - HGF 40ng/ml
10 20 0 60 40 10 40 5 10 120 5 0 120 20 120 0 60 5 60 20 time [min]
p S6
total S6
