# Supplementary material for: Basal MET phosphorylation is an indicator of hepatocyte dysregulation in liver disease
Source: Mol Syst Biol. 2024 Jan 12;20(3):187–216. doi: 10.1038/s44320-023-00007-4 (PMC10912216; doi:10.1038/s44320-023-00007-4)
Supplement: Supplementary file 9 — Source Data Fig. 2 [file 44320_2023_7_MOESM9_ESM.zip › Figure 2/2C/Gel4-2_5-2_6-2_B2_tMet.pdf]

Gel4-2:

WD	SD	SD	WD	WD	SD	WD	SD	SD	SD	WD	WD	WD	SD	WD	SD	WD	WD	SD	SD	WD	diet
M3	M2	M2	M3	M3	M2	M3	M2	M2	M2	M3	M3	M3	M2	M3	M2	M3	M3	M2	M2	M3	replicate
+	-	-	+	+	-	+	-	-	-	+	+	+	-	+	-	+	+	-	-	+	HGF 40ng/ml
60	0	4h	120	0	18h	20	40	24h	5	5	18h	40	10	4h	120	24h	10	3h	20	3h	time [min]

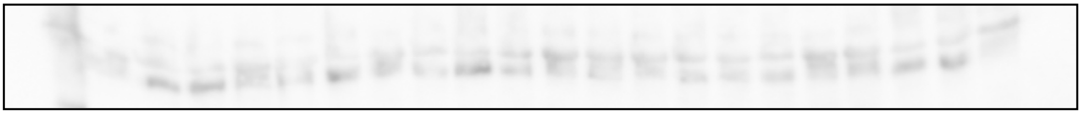

total Met (lower band)

Gel 5-2:

WD	SD	WD	WD	SD	WD	SD	SD	WD	WD	WD	SD	WD	SD	WD	WD	SD	SD	WD	diet
M2	M3	M2	M2	M3	M2	M3	M3	M2	M2	M2	M3	M2	M3	M2	M2	M3	M3	M2	replicate
-	-	-	-	-	-	-	-	-	-	-	-	-	-	-	-	-	-	-	HGF 40ng/ml
60	0	120	0	18h	20	24h	5	5	18h	40	10	4h	120	24h	10	3h	20	3h	time [min]

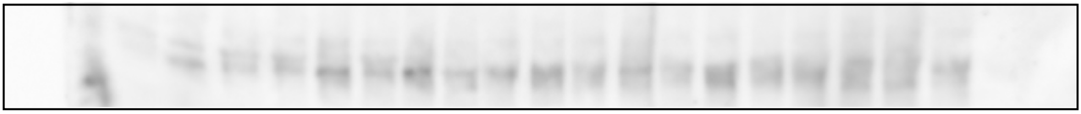

total Met (lower band)

Gel 6-2:

WD	WD	WD	WD	WD	WD	WD	WD	WD	WD	WD	WD	WD	WD	WD	WD	WD	WD	WD	WD	WD	WD
M2	M3	M3	M2	M2	M3	M3	M2	M3	M3	M3	M2	M2	M2	M3	M2	M3	M2	M2	M3	M3	M2
+	-	-	+	+	-	-	+	-	-	-	+	+	+	-	+	-	+	+	-	-	+
60	0	4h	120	0	18h	60	20	40	24h	5	5	18h	40	10	4h	120	24h	10	3h	20	3h

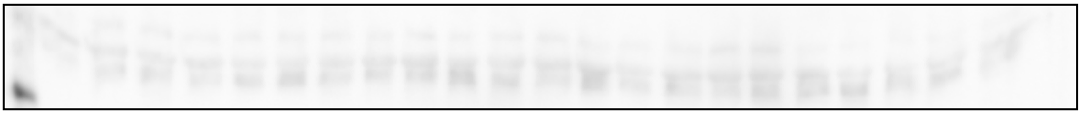

total Met (lower band)
