# Supplementary material for: Basal MET phosphorylation is an indicator of hepatocyte dysregulation in liver disease
Source: Mol Syst Biol. 2024 Jan 12;20(3):187–216. doi: 10.1038/s44320-023-00007-4 (PMC10912216; doi:10.1038/s44320-023-00007-4)
Supplement: Supplementary file 9 — Source Data Fig. 2 [file 44320_2023_7_MOESM9_ESM.zip › Figure 2/2C/Gel4-2_B1_pMet_tMet_pAktT308.pdf]

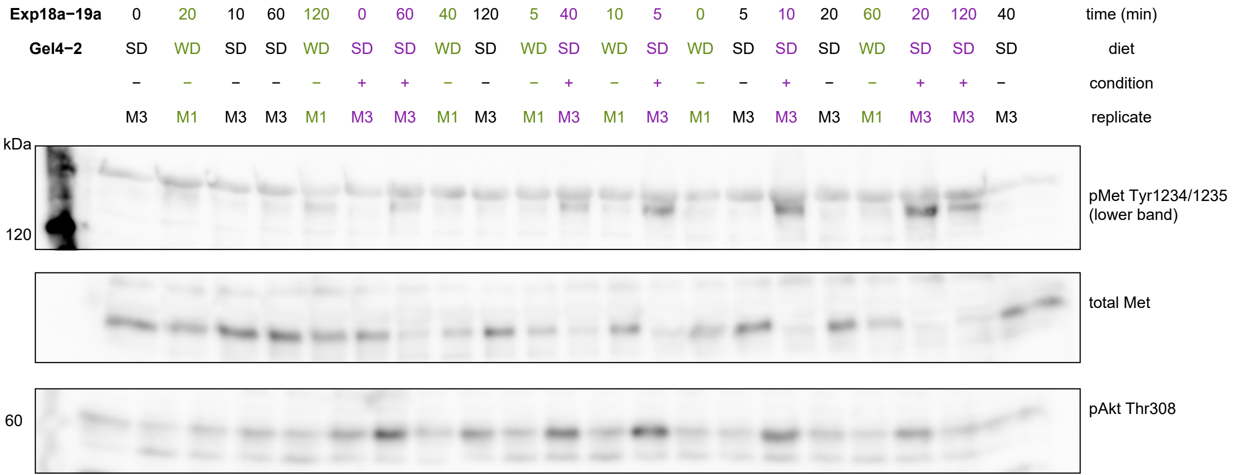
Exp18a−19a
0 20 10 60 120 0 60 40 120 5 40 10 5 0 5 10 20 60 20 120 40
time (min)
Gel4−2
SD WD SD SD WD SD SD WD SD WD SD WD SD WD SD SD SD WD SD SD SD
diet
− − − − − + + − − − + − + − − + − − + + −
condition
M3 M1 M3 M3 M1 M3 M3 M1 M3 M1 M3 M1 M3 M1 M3 M3 M3 M1 M3 M3 M3
replicate
kDa
120
pMet Tyr1234/1235
(lower band)
total Met
60
pAkt Thr308
