# Supplementary material for: Basal MET phosphorylation is an indicator of hepatocyte dysregulation in liver disease
Source: Mol Syst Biol. 2024 Jan 12;20(3):187–216. doi: 10.1038/s44320-023-00007-4 (PMC10912216; doi:10.1038/s44320-023-00007-4)
Supplement: Supplementary file 9 — Source Data Fig. 2 [file 44320_2023_7_MOESM9_ESM.zip › Figure 2/2C/Gel4-2_B3a_pMet_tMet_pAktT308.pdf]

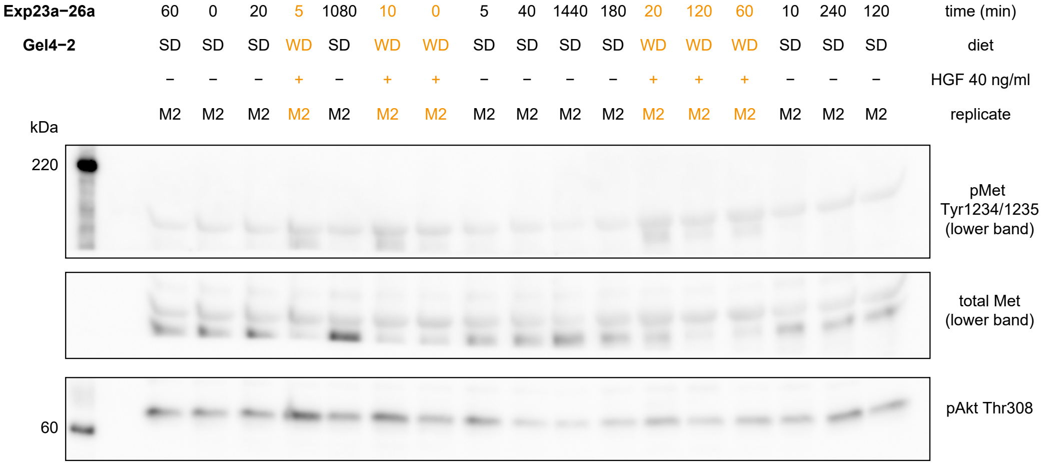
Exp23a−26a
Gel4−2
60 0 20 5 1080 10 0 5 40 1440 180 20 120 60 10 240 120
time (min)
SD SD SD WD SD WD WD SD SD SD SD WD WD WD SD SD SD
diet
− − − + − + + − − − − + + + − − −
HGF 40 ng/ml
M2 M2 M2 M2 M2 M2 M2 M2 M2 M2 M2 M2 M2 M2 M2 M2 M2
replicate
kDa
220
pMet Tyr1234/1235 (lower band)
total Met (lower band)
60
pAkt Thr308
