# Supplementary material for: Basal MET phosphorylation is an indicator of hepatocyte dysregulation in liver disease
Source: Mol Syst Biol. 2024 Jan 12;20(3):187–216. doi: 10.1038/s44320-023-00007-4 (PMC10912216; doi:10.1038/s44320-023-00007-4)
Supplement: Supplementary file 9 — Source Data Fig. 2 [file 44320_2023_7_MOESM9_ESM.zip › Figure 2/2C/Gel4_B1_pS6_tS6.pdf]

Membrane 4:

SD	SD	WD	SD	WD	SD	SD	SD	SD	SD	WD	SD	SD	WD	SD	WD	SD	**WD**	SD	SD	diet
M3	M3	M1	M3	M1	M3	M3	M3	M3	M3	M1	M3	M3	M1	M3	M1	M3	**M3**	M3	M3	replicate
+	-	-	+	-	-	+	-	+	-	-	+	-	-	-	-	+	**-**	-	+	HGF 40ng/ml
60	5	0	10	20	60	20	120	0	20	40	120	0	5	40	10	5	**120**	10	40	time [min]

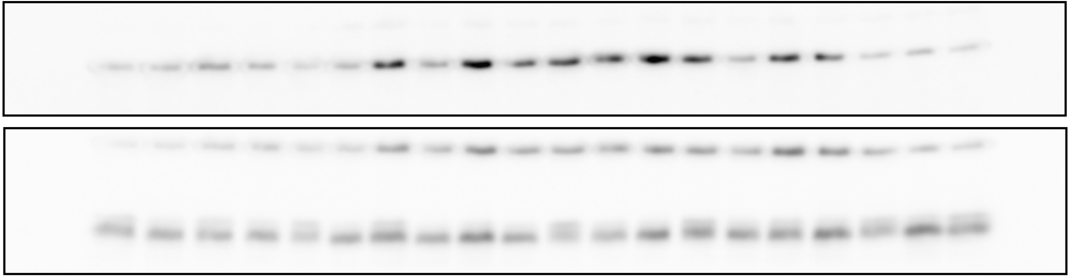

p S6

total S6
