# Supplementary material for: Basal MET phosphorylation is an indicator of hepatocyte dysregulation in liver disease
Source: Mol Syst Biol. 2024 Jan 12;20(3):187–216. doi: 10.1038/s44320-023-00007-4 (PMC10912216; doi:10.1038/s44320-023-00007-4)
Supplement: Supplementary file 9 — Source Data Fig. 2 [file 44320_2023_7_MOESM9_ESM.zip › Figure 2/2C/Gel5-2_B3a_pMet_tMet_pAktT308.pdf]

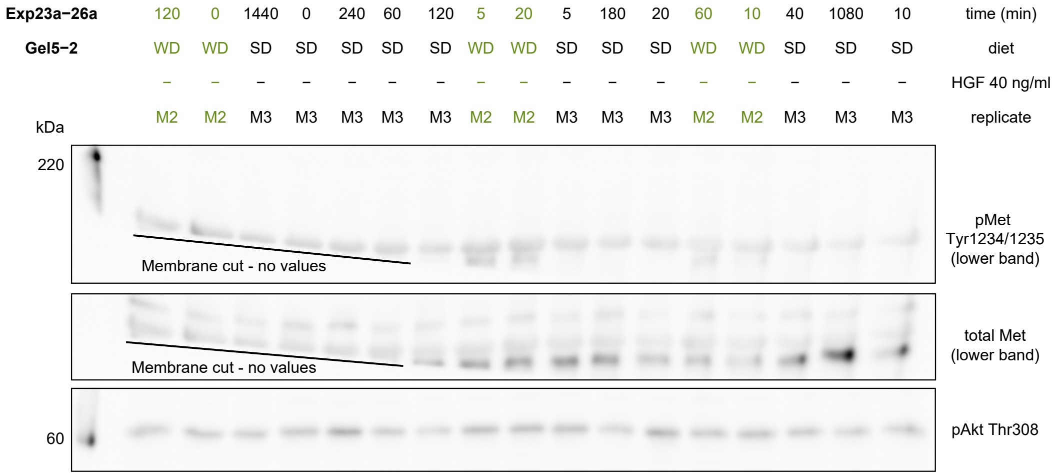

Exp23a−26a
Gel5−2
120 0 1440 0 240 60 120 5 20 5 180 20 60 10 40 1080 10
time (min)
WD WD SD SD SD SD SD WD WD SD SD SD WD WD SD SD SD
diet
– – – – – – – – – – – – – – – – –
HGF 40 ng/ml
M2 M2 M3 M3 M3 M3 M3 M2 M2 M3 M3 M3 M2 M2 M3 M3 M3
replicate
kDa
220
60
Membrane cut - no values
pMet Tyr1234/1235 (lower band)
Membrane cut - no values
total Met (lower band)
pAkt Thr308
