# Supplementary material for: Basal MET phosphorylation is an indicator of hepatocyte dysregulation in liver disease
Source: Mol Syst Biol. 2024 Jan 12;20(3):187–216. doi: 10.1038/s44320-023-00007-4 (PMC10912216; doi:10.1038/s44320-023-00007-4)
Supplement: Supplementary file 9 — Source Data Fig. 2 [file 44320_2023_7_MOESM9_ESM.zip › Figure 2/2C/Gel5_Gel6_B2_pAkt_tAkt_pERK_tERK.pdf]

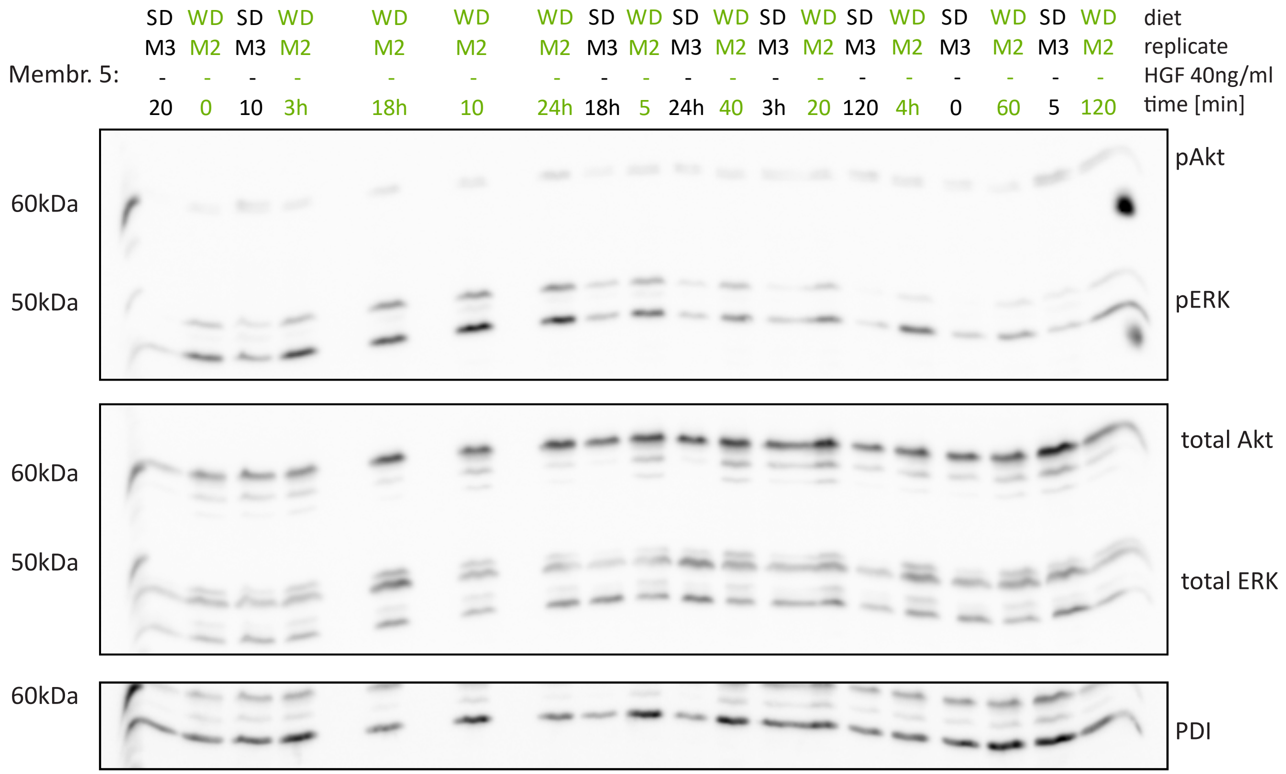
Membr. 5:
SD WD SD WD WD WD WD SD WD SD WD SD WD SD WD SD WD SD WD diet
M3 M2 M3 M2 M2 M2 M2 M3 M2 M3 M2 M3 M2 M3 M2 M3 M2 M3 M2 replicate
- - - - - - - - - - - - - - - - - - - HGF 40ng/ml
20 0 10 3h 18h 10 24h 18h 5 24h 40 3h 20 120 4h 0 60 5 120 time [min]
60kDa
50kDa
60kDa
50kDa
60kDa
pAkt
pERK
total Akt
total ERK
PDI

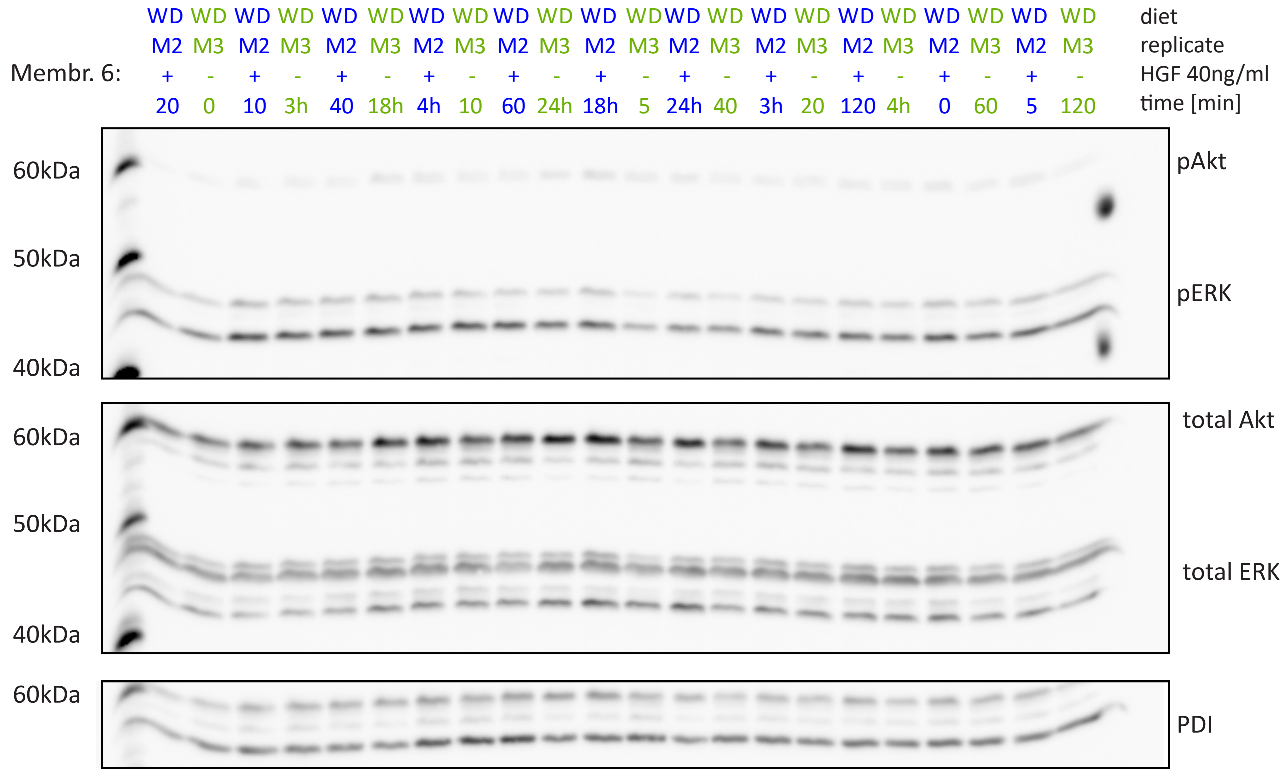
Membr. 6:
WD WD WD WD WD WD WD WD WD WD WD WD WD WD WD WD WD WD WD WD WD WD diet
M2 M3 M2 M3 M2 M3 M2 M3 M2 M3 M2 M3 M2 M3 M2 M3 M2 M3 M2 M3 M2 M3 replicate
+ - + - + - + - + - + - + - + - + - + - + - HGF 40ng/ml
20 0 10 3h 40 18h 4h 10 60 24h 18h 5 24h 40 3h 20 120 4h 0 60 5 120 time [min]
60kDa
50kDa
40kDa
60kDa
50kDa
40kDa
60kDa
pAkt
pERK
total Akt
total ERK
PDI
