# Supplementary material for: Basal MET phosphorylation is an indicator of hepatocyte dysregulation in liver disease
Source: Mol Syst Biol. 2024 Jan 12;20(3):187–216. doi: 10.1038/s44320-023-00007-4 (PMC10912216; doi:10.1038/s44320-023-00007-4)
Supplement: Supplementary file 12 — Source Data Fig. 6 [file 44320_2023_7_MOESM12_ESM.zip › Figure 6/6A/PHH_Western Blots_pAkt_pERK_tAkt_tERK.pdf]

pAkt pERK

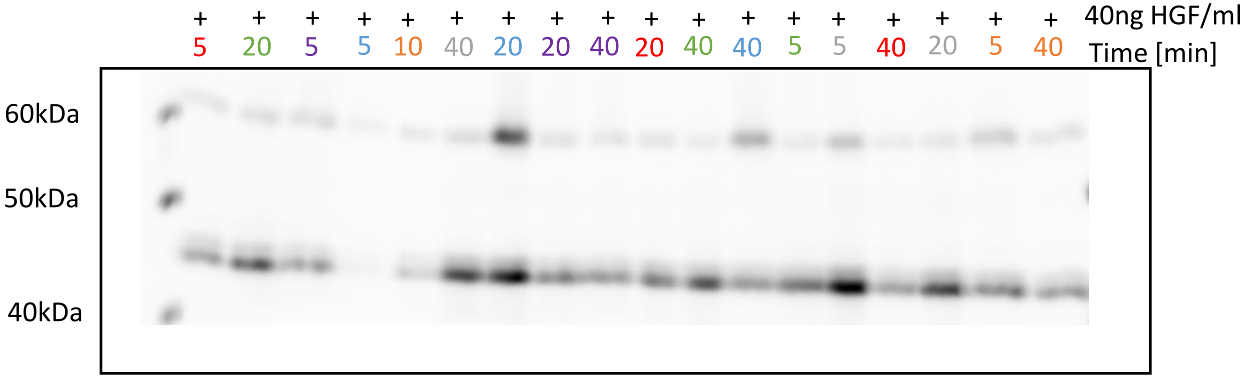

tAkt tERK

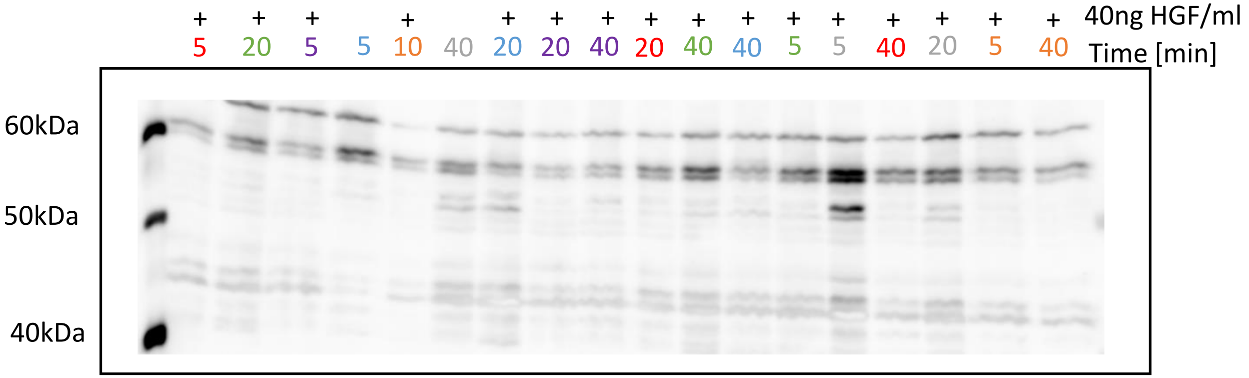

Exp.2 -> Patient 5
Exp.3
Exp.4 -> Patient 6
Exp.6 -> Patient 7
Exp.9
Exp.11 -> Patient 1

pAkt pERK

+	+	+	+	+	+	+	+	+	+	+	+	+	+	+	40ng HGF/ml
20	5	40	5	5	20	40	5	20	5	40	40	40	20	20	Time [min]

60kDa

50kDa

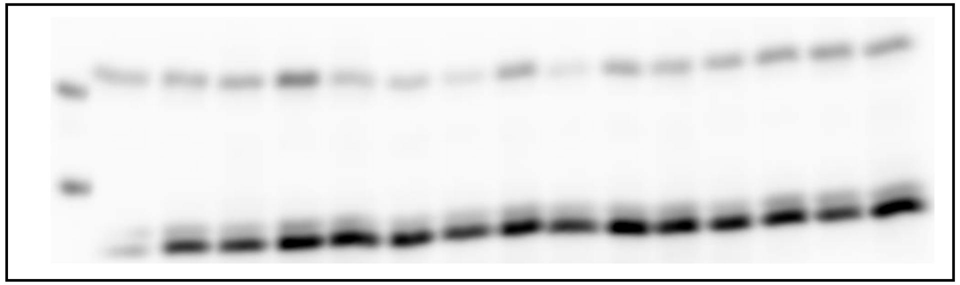

tAkt tERK

+	+	+	+	+	+	+	+	+	+	+	+	+	+	+	40ng HGF/ml
20	5	40	5	5	20	40	5	20	5	40	40	40	20	20	Time [min]

60kDa

50kDa

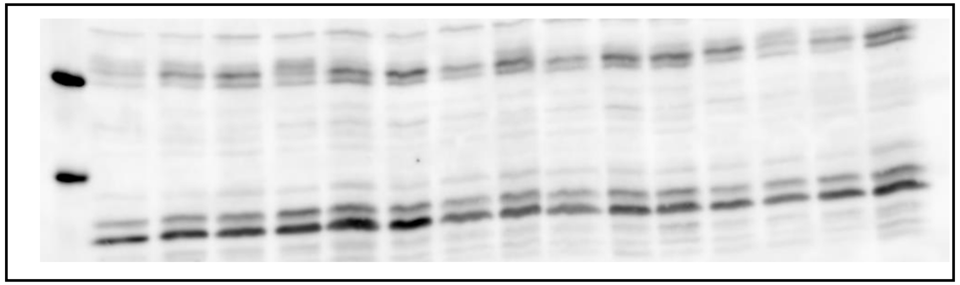

Exp.10

Exp.11 -> Patient 1

Exp.12 -> Patient 2

Exp.13 -> Patient 3

Exp.15 -> Patient 4
