# Supplementary material for: Basal MET phosphorylation is an indicator of hepatocyte dysregulation in liver disease
Source: Mol Syst Biol. 2024 Jan 12;20(3):187–216. doi: 10.1038/s44320-023-00007-4 (PMC10912216; doi:10.1038/s44320-023-00007-4)
Supplement: Supplementary file 12 — Source Data Fig. 6 [file 44320_2023_7_MOESM12_ESM.zip › Figure 6/6A/PHH_Western Blots_pMet_tMet.pdf]

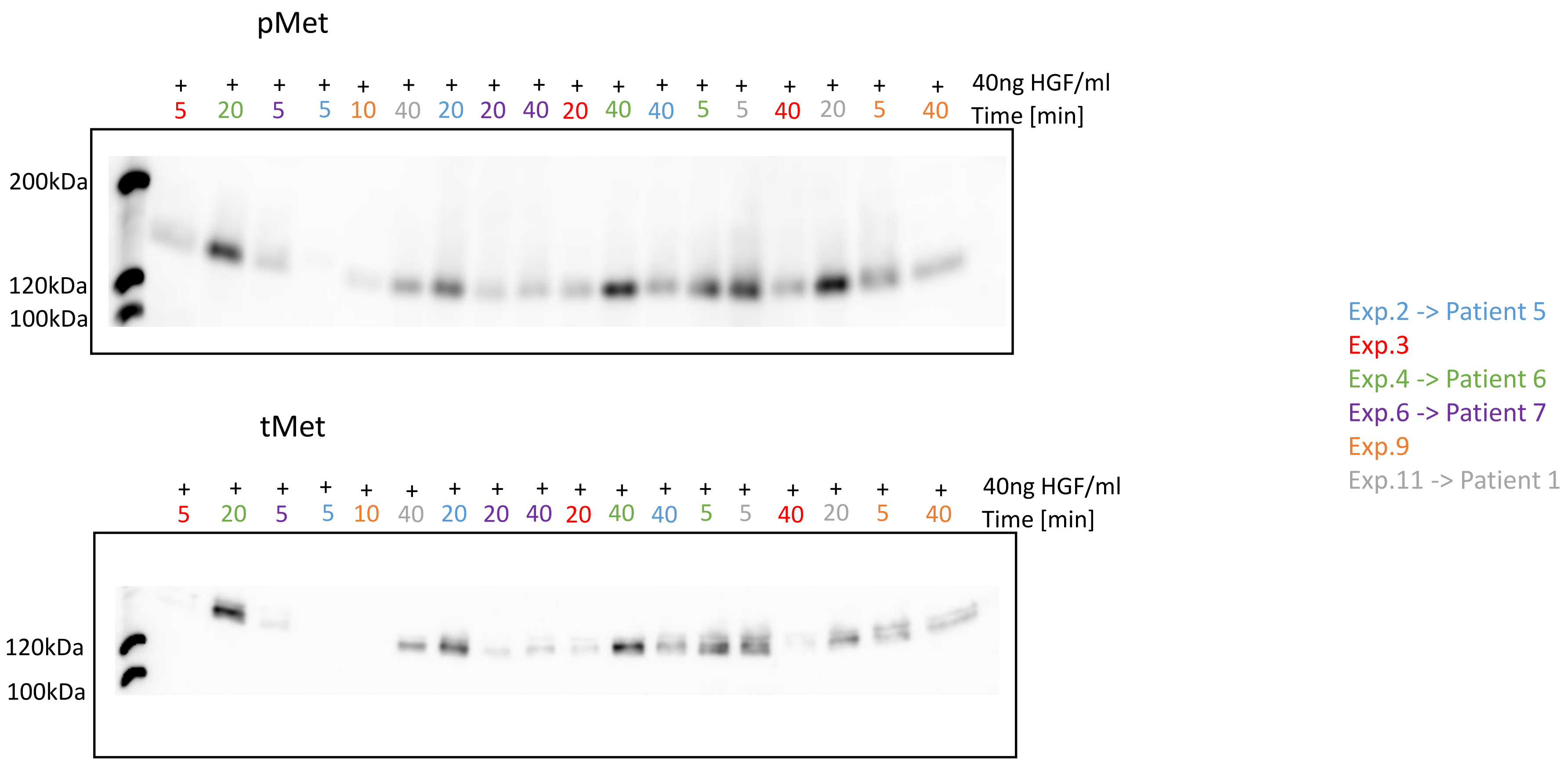
pMet
+ + + + + + + + + + + + + + + + + + 40ng HGF/ml
5 20 5 5 10 40 20 20 40 20 40 40 5 5 40 20 5 40 Time [min]
200kDa
120kDa
100kDa
tMet
+ + + + + + + + + + + + + + + + + + 40ng HGF/ml
5 20 5 5 10 40 20 20 40 20 40 40 5 5 40 20 5 40 Time [min]
120kDa
100kDa
Exp.2 -> Patient 5
Exp.3
Exp.4 -> Patient 6
Exp.6 -> Patient 7
Exp.9
Exp.11 -> Patient 1

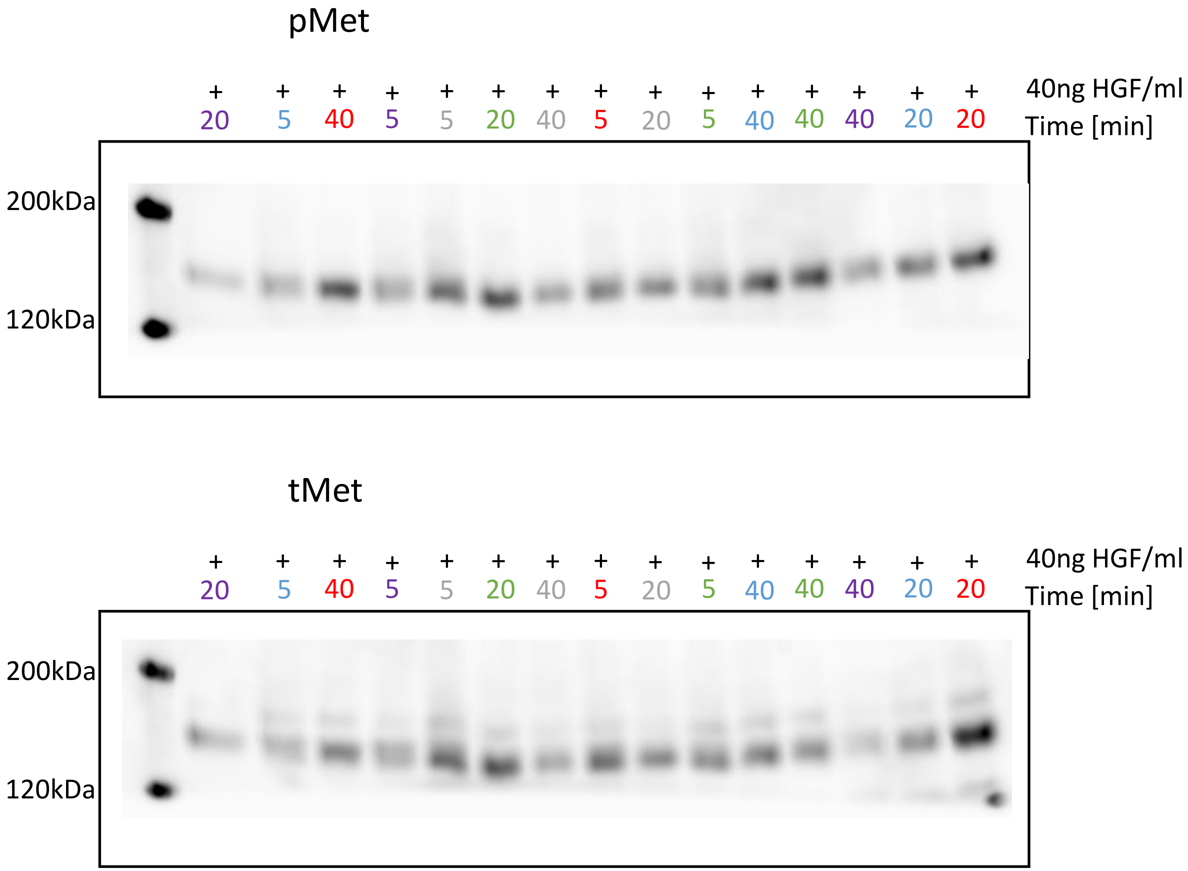

Exp.10
Exp.11 -> Patient 1
Exp.12 -> Patient 2
Exp.13 -> Patient 3
Exp.15 -> Patient 4
