# Supplementary material for: Basal MET phosphorylation is an indicator of hepatocyte dysregulation in liver disease
Source: Mol Syst Biol. 2024 Jan 12;20(3):187–216. doi: 10.1038/s44320-023-00007-4 (PMC10912216; doi:10.1038/s44320-023-00007-4)
Supplement: Supplementary file 12 — Source Data Fig. 6 [file 44320_2023_7_MOESM12_ESM.zip › Figure 6/6A/PHH_Western Blots_pS6K_tS6K.pdf]

pS6 Kinase

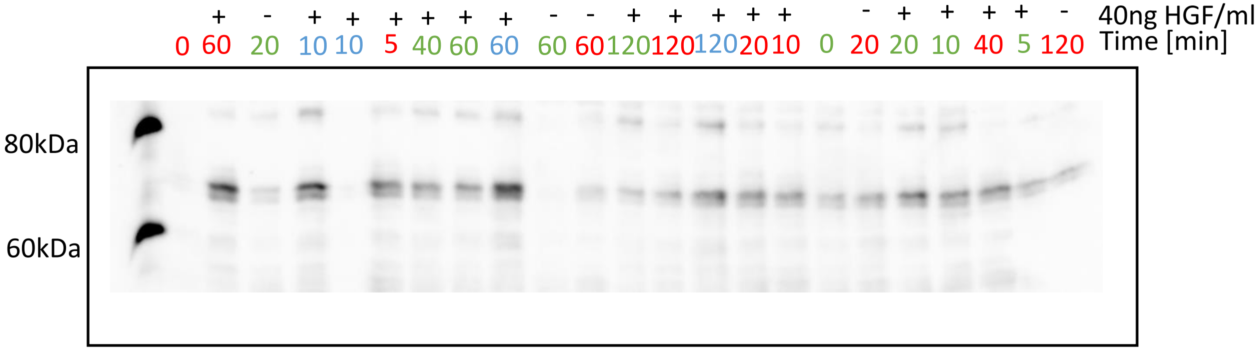


Exp.2 -> Patient 5

Exp.3

Exp.4 -> Patient 6

S6 Kinase

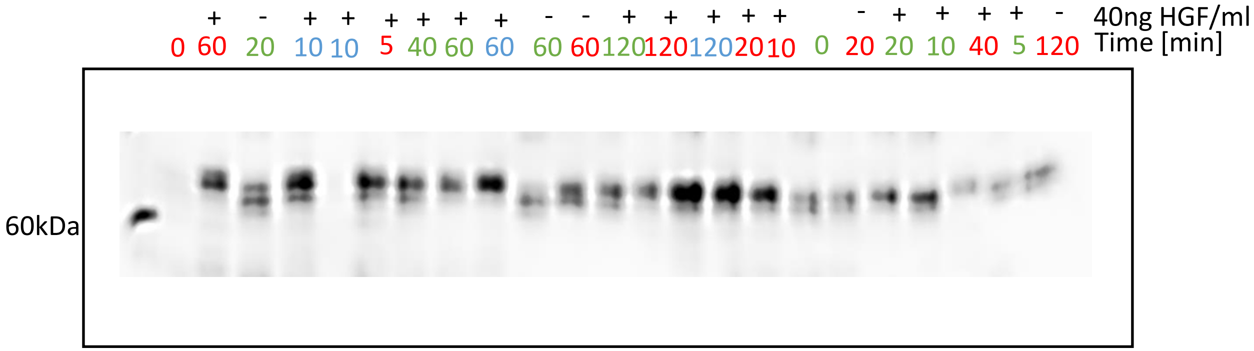

pS6 Kinase

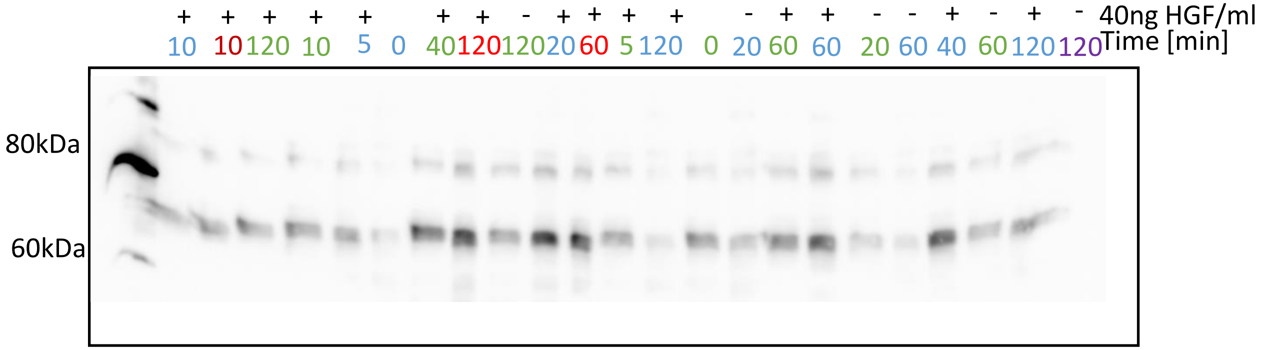


S6 Kinase

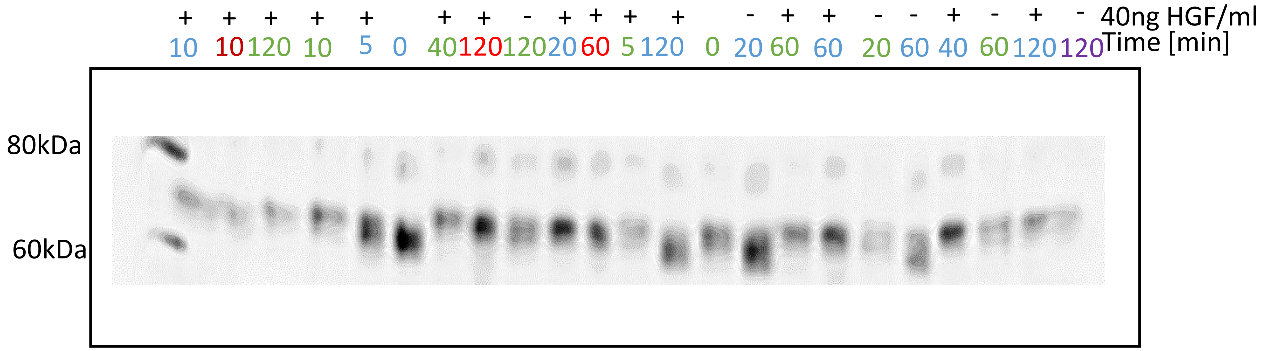


Exp.3

Exp.4 -> Patient 6

Exp.9

Exp.10

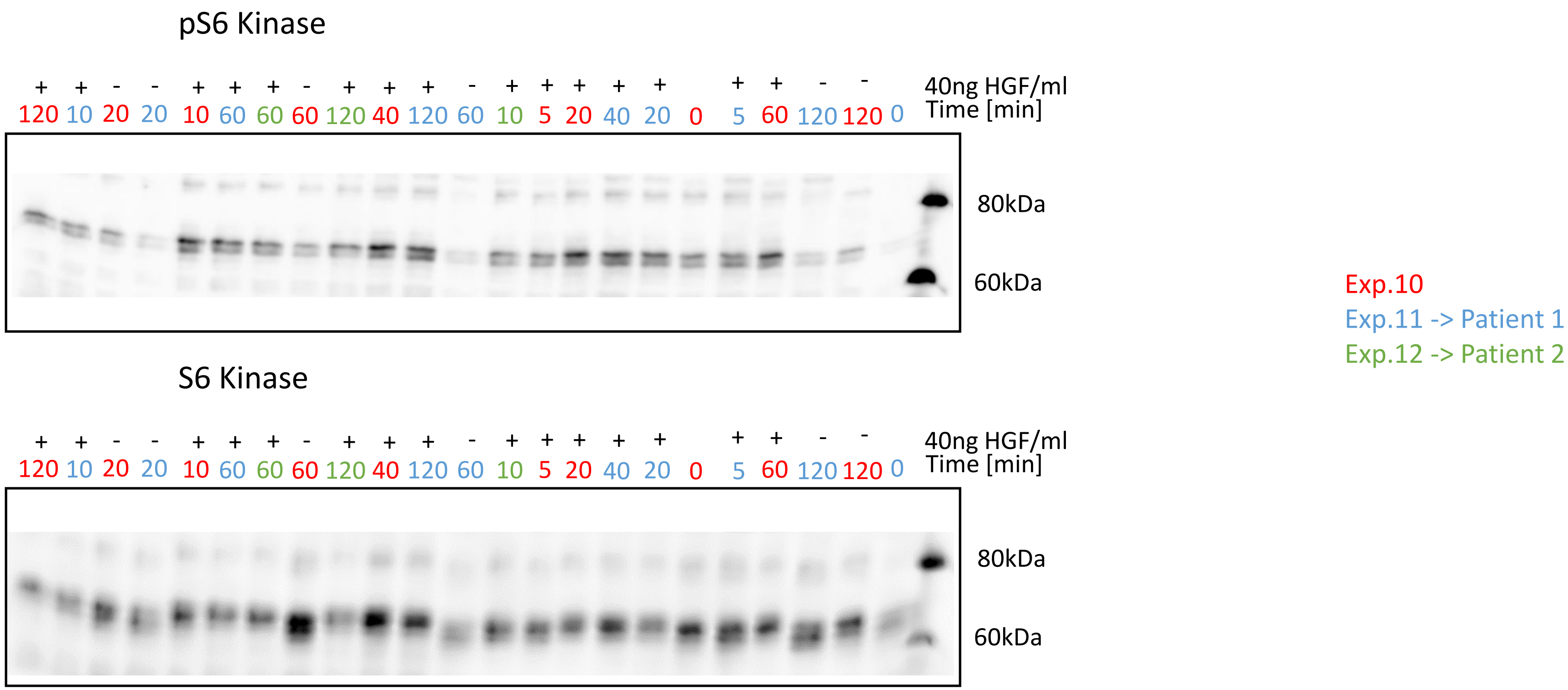

pS6 Kinase
+ + - - + + + - + + + - + + + + + + + - -
40ng HGF/ml
120 10 20 20 10 60 60 60 120 40 120 60 10 5 20 40 20 0 5 60 120 120 0
Time [min]
80kDa
60kDa
S6 Kinase
+ + - - + + + - + + + - + + + + + + + - -
40ng HGF/ml
120 10 20 20 10 60 60 60 120 40 120 60 10 5 20 40 20 0 5 60 120 120 0
Time [min]
80kDa
60kDa
Exp.10
Exp.11 -> Patient 1
Exp.12 -> Patient 2

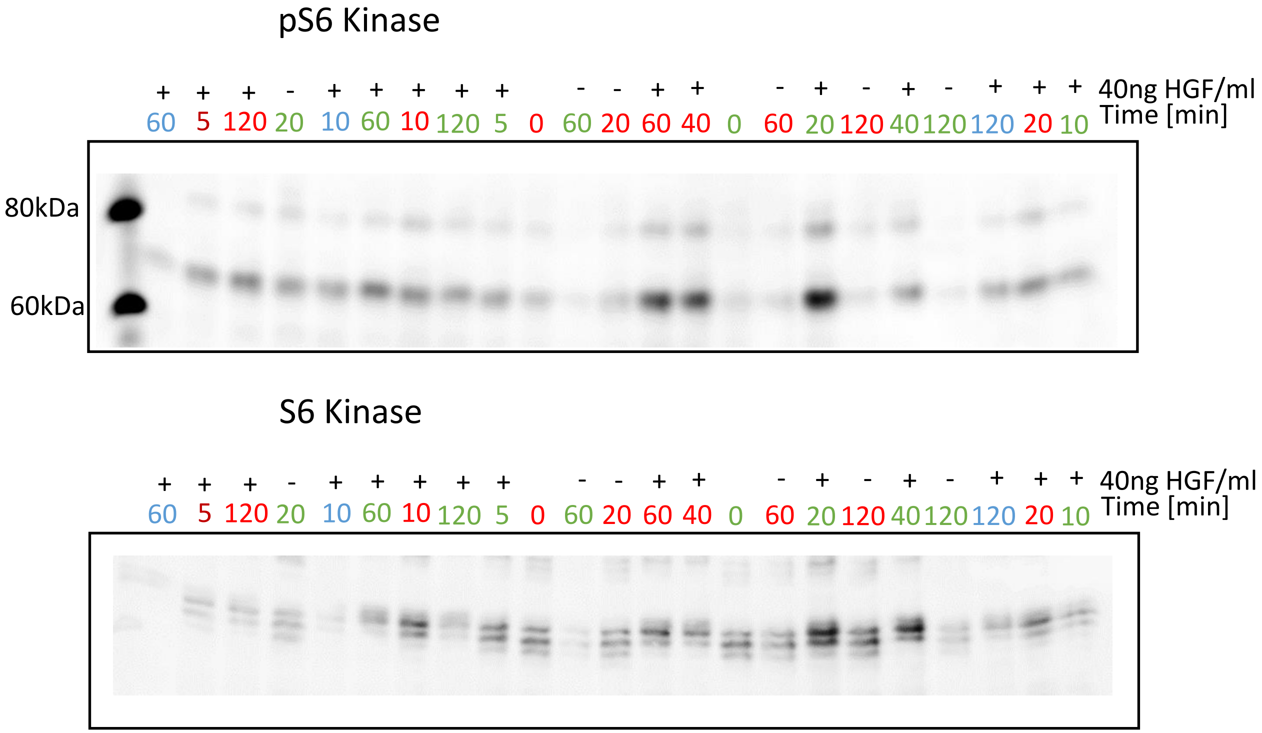


Exp.12 -> Patient 2

Exp.13 -> Patient 3

Exp.15 -> Patient 4

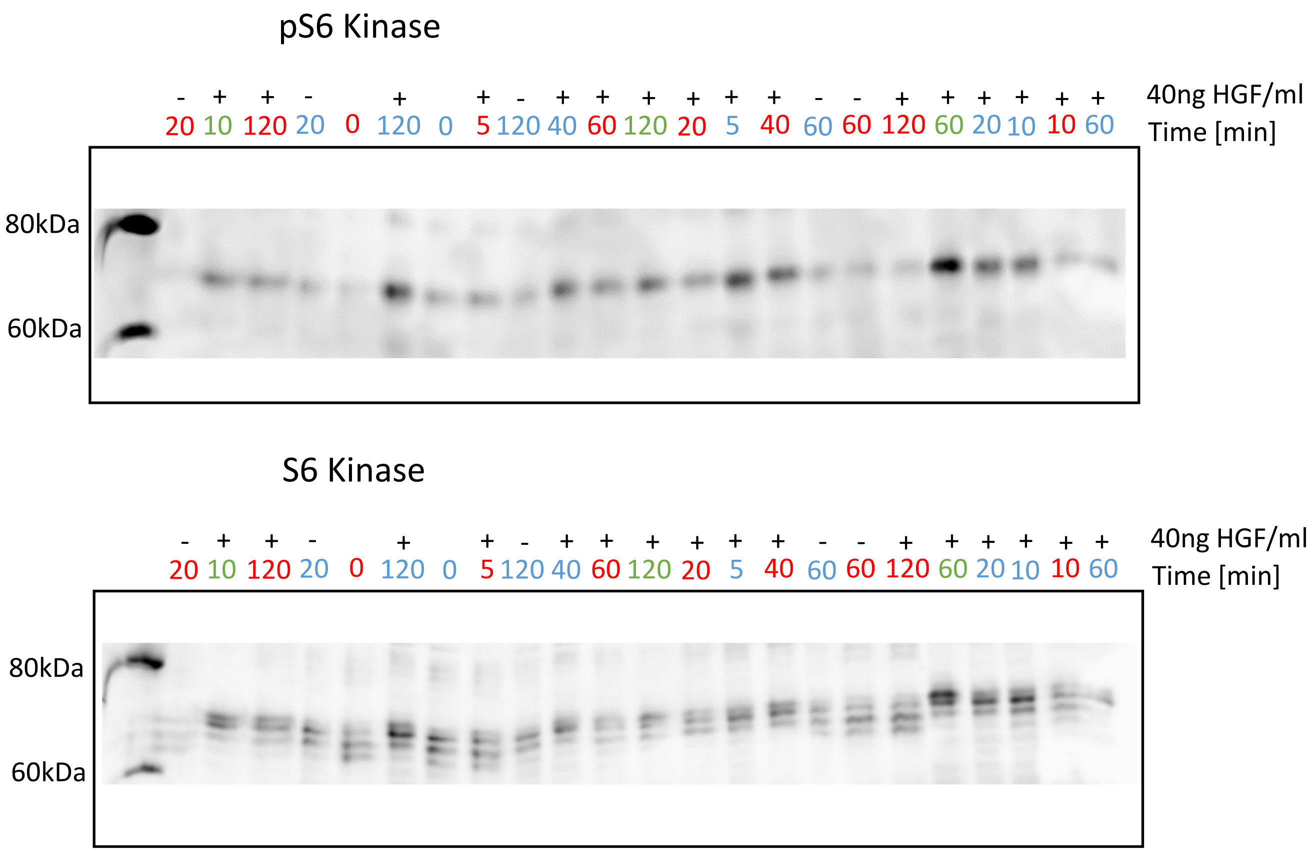


Exp.15 -> Patient 4
Exp.6 -> Patient 7
Exp.2 -> Patient 5
