# Supplementary material for: Basal MET phosphorylation is an indicator of hepatocyte dysregulation in liver disease
Source: Mol Syst Biol. 2024 Jan 12;20(3):187–216. doi: 10.1038/s44320-023-00007-4 (PMC10912216; doi:10.1038/s44320-023-00007-4)
Supplement: Supplementary file 14 — Expanded View Figures [file 44320_2023_7_MOESM14_ESM.pdf]

Expanded View Figures

Figure EV1. Quantitative data for calibration of the mouse model.

(**A**) Model calibration with HGF dose-resolved signal transduction measurements in primary mouse hepatocytes of SD and WD mice. Cells were stimulated with indicated doses of HGF for 10 min and phosphorylation of MET, ERK and AKT was quantified by immunoblotting. Signal is shown in arbitrary units (a.u.). Data points are displayed as dots with error bars representing 1σ confidence interval estimated from biological replicates ($n = 3$–9 per diet and dose) using a combined scaling and error model. Model trajectories are represented by solid lines. (**B**) Model calibration with HGF time-resolved signal transduction measurements in primary mouse hepatocytes of SD and WD mice. Immunoblot measurements for ERK, AKT and S6 abundance upon stimulation with 40 ng/ml HGF. Data points are displayed as dots along with error bars representing 1σ confidence interval estimated from biological replicates ($n = 3$–9 per diet and time point) using a combined scaling and error model. Model trajectories are depicted as solid lines. (**C**) A Bayesian information criterion (BIC) analysis was performed to determine the diet-specific parameters needed to describe the experimental data. The threshold for rejection was set to ΔBIC = 10 as suggested (Lorah and Womack, 2019). H0 including 64 parameters could be reduced to H2.1.2 including 61 parameters, suggesting that only the basal phosphorylation rate of the HGF receptor MET was dysregulated between diets. (**D**) Correlation of basal MET phosphorylation and MET abundance at time point 0 h. Dots display the respective values for each mouse ($n = 9$ per diet). Correlation coefficient and p value were calculated using a simple linear regression (p value = 0.19).

© The Author(s)

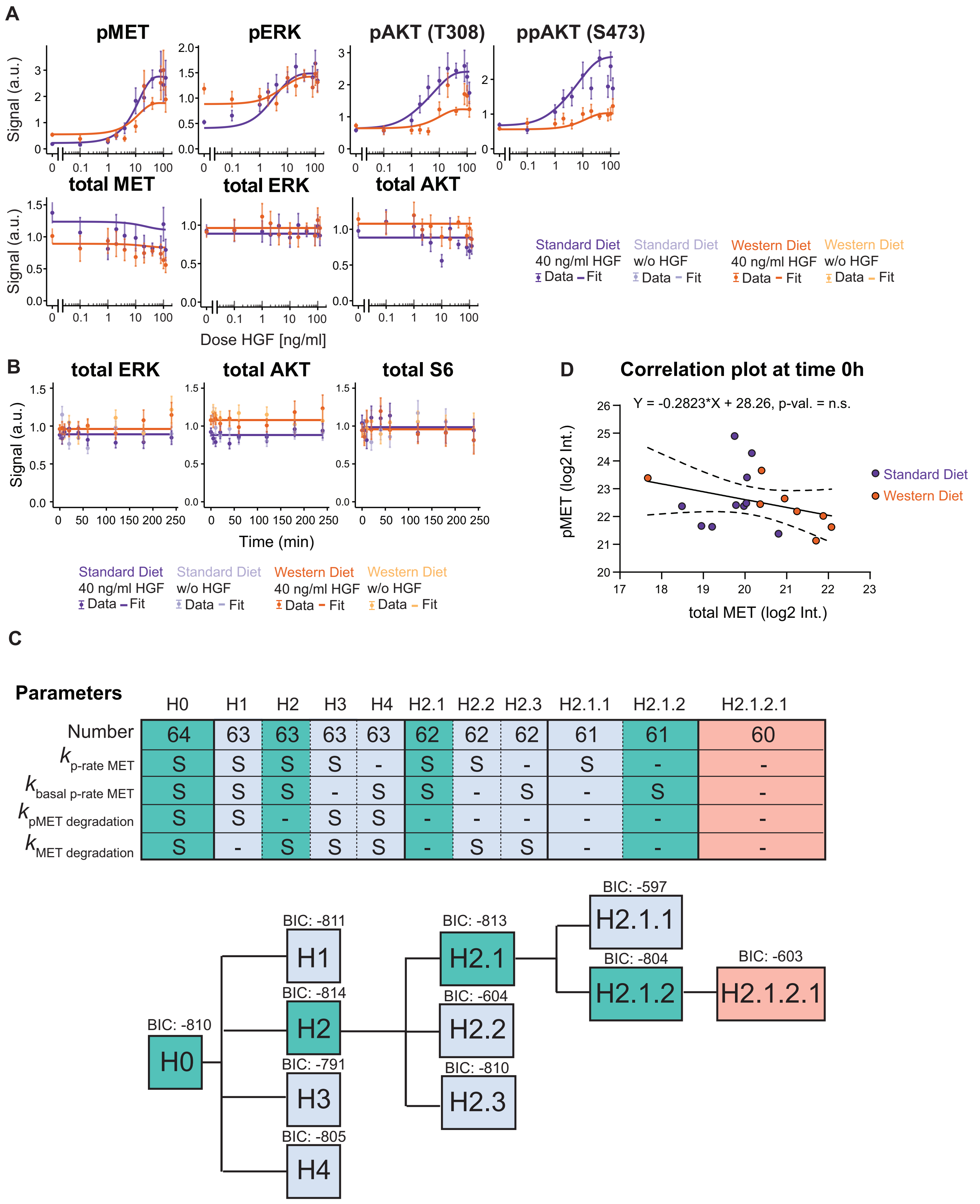
A
pMET
pERK
pAKT (T308)
ppAKT (S473)
total MET
total ERK
total AKT
Signal (a.u.)
Dose HGF [ng/ml]
Standard Diet 40 ng/ml HGF Data Fit
Standard Diet w/o HGF Data Fit
Western Diet 40 ng/ml HGF Data Fit
Western Diet w/o HGF Data Fit
B
total ERK
total AKT
total S6
Signal (a.u.)
Time (min)
D
Correlation plot at time 0h
Y = -0.2823*X + 28.26, p-val. = n.s.
pMET (log2 Int.)
total MET (log2 Int.)
Standard Diet
Western Diet
C
Parameters
H0 H1 H2 H3 H4 H2.1 H2.2 H2.3 H2.1.1 H2.1.2 H2.1.2.1
Number 64 63 63 63 63 62 62 62 61 61 60
k p-rate MET S S S S - S S - S - -
k basal p-rate MET S S S - S S - S - S -
k pMET degradation S S - S S - - - - - -
k MET degradation S - S S S - S S - - -
BIC: -810 H0
BIC: -811 H1
BIC: -814 H2
BIC: -791 H3
BIC: -805 H4
BIC: -813 H2.1
BIC: -604 H2.2
BIC: -810 H2.3
BIC: -597 H2.1.1
BIC: -804 H2.1.2
BIC: -603 H2.1.2.1

 © The Author(s)

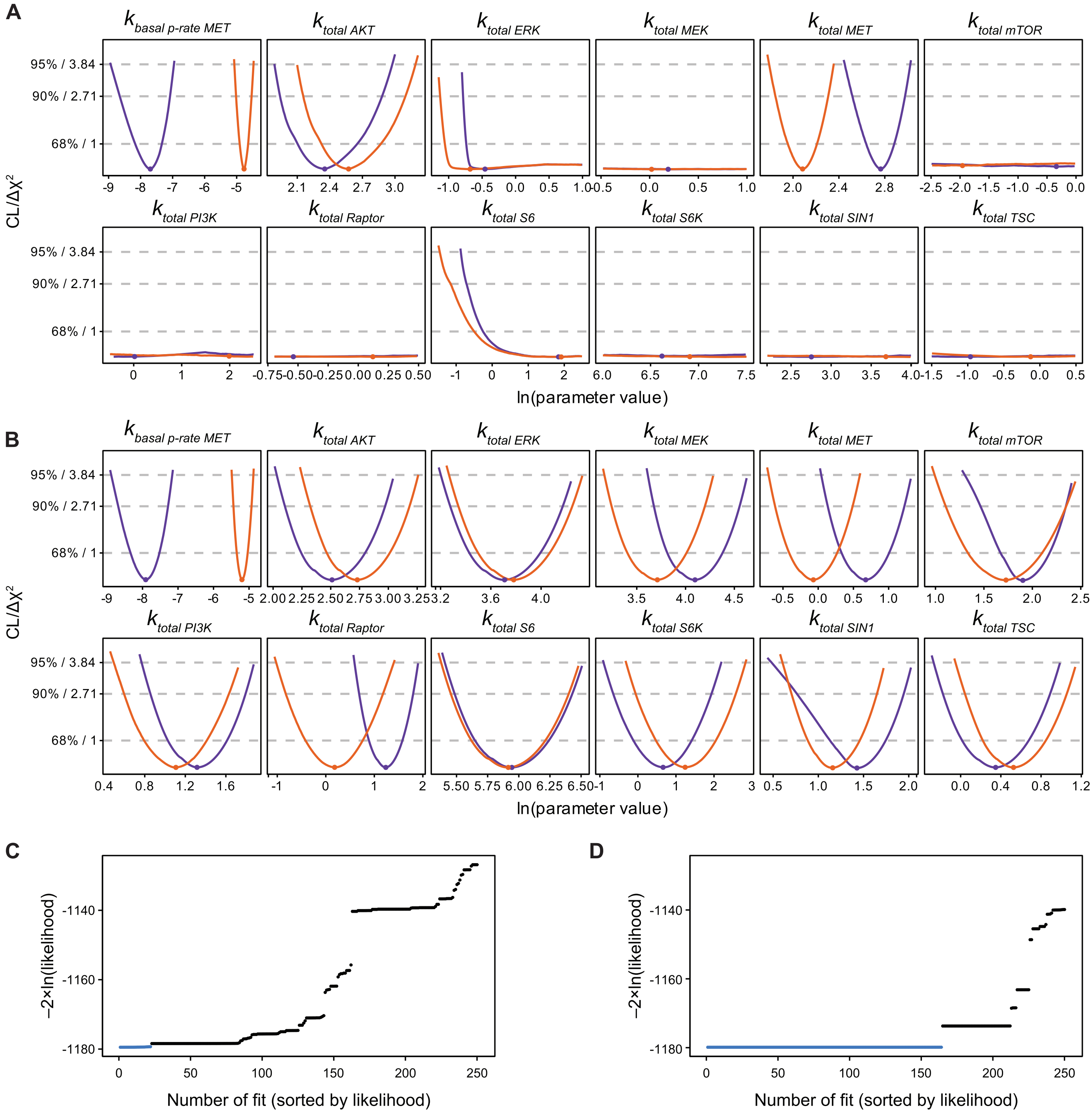

Figure EV2. Impact of the DIA data on identifiability and convergence.

(**A**) The profile likelihood as a measure of parameter identifiability (Raue et al, 2009) is depicted for all dysregulated parameters before implementation of the DIA data. If the negative log likelihood reaches a statistical threshold in both directions, the parameter has defined confidence bounds and is therefore called identifiable. If this limit is not reached on both sides, the parameter is classified as unidentifiable. Solid lines indicate the profile likelihood of dysregulated parameters for SD (purple) and WD (orange) along with the optimal parameter values as dots. Dashed lines depict thresholds for the confidence interval assessment. (**B**) The profile likelihood as a measure of parameter identifiability is depicted for all dysregulated parameters after implementation of the DIA data. (**C**) The convergence of the optimization before implementation of the DIA data is assessed based on a waterfall plot (Raue et al, 2013). This plot depicts the results of 250 optimization runs starting from randomly selected parameter sets sorted by the negative log likelihood. The global optimum, indicated in blue, was reached in 22 of the 250 cases. (**D**) The convergence of the optimization after implementation of the DIA data is assessed based on a waterfall plot. The global optimum, indicated in blue, was reached in 164 out of 250 cases.

© The Author(s)

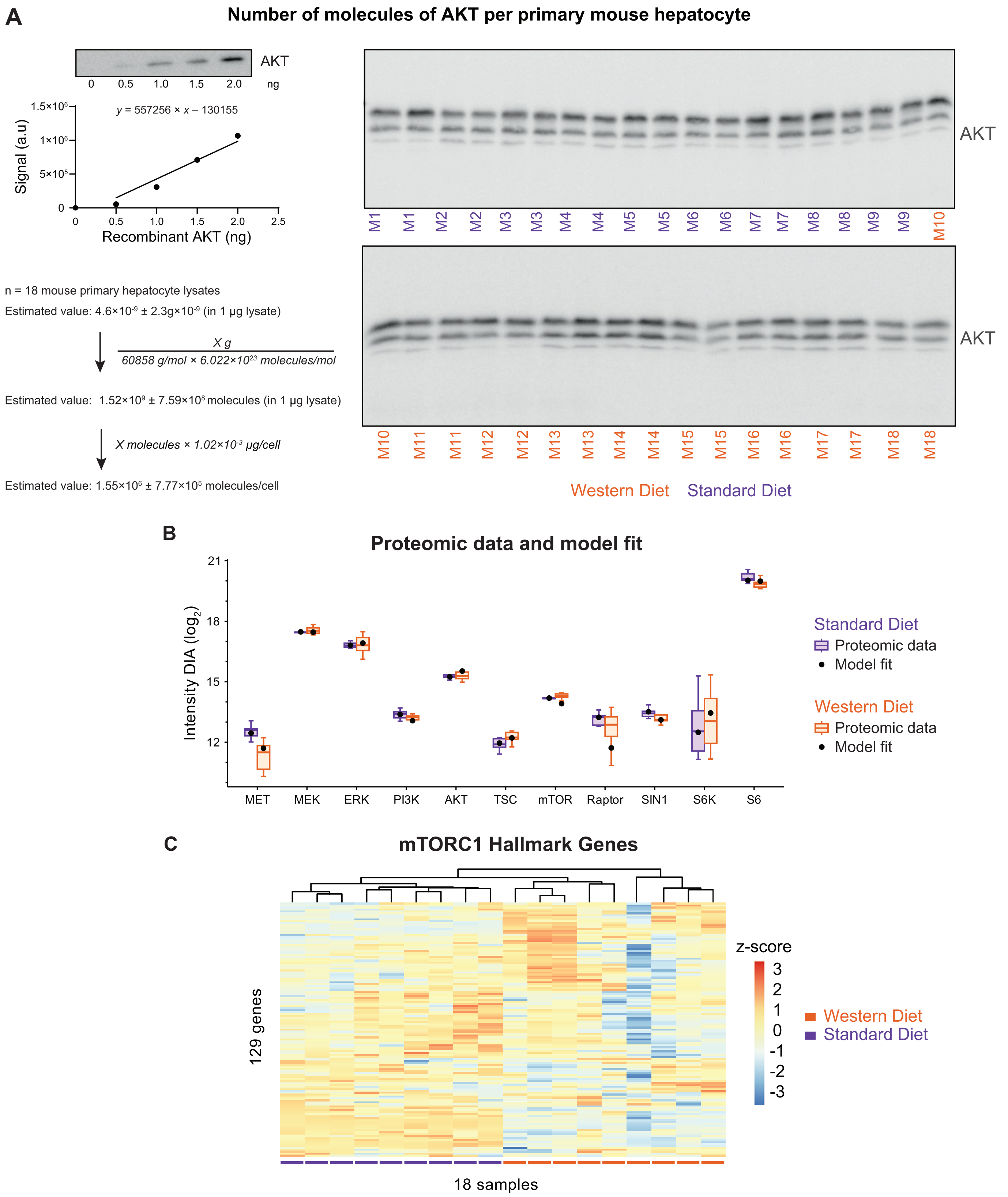
A
Number of molecules of AKT per primary mouse hepatocyte
AKT
0 0.5 1.0 1.5 2.0 ng
y = 557256 × x – 130155
Signal (a.u)
1.5×10^6
1×10^6
5×10^5
0
0 0.5 1.0 1.5 2.0 2.5
Recombinant AKT (ng)
n = 18 mouse primary hepatocyte lysates
Estimated value: 4.6×10^-9 ± 2.3g×10^-9 (in 1 µg lysate)
X g / 60858 g/mol × 6.022×10^23 molecules/mol
Estimated value: 1.52×10^9 ± 7.59×10^8 molecules (in 1 µg lysate)
X molecules × 1.02×10^-3 µg/cell
Estimated value: 1.55×10^6 ± 7.77×10^5 molecules/cell
AKT
M1 M1 M2 M2 M3 M3 M4 M4 M5 M5 M6 M6 M7 M7 M8 M8 M9 M9 M10
AKT
M10 M11 M11 M12 M12 M13 M13 M14 M14 M15 M15 M16 M16 M17 M17 M18 M18
Western Diet
Standard Diet
B
Proteomic data and model fit
Intensity DIA (log2)
21
18
15
12
MET MEK ERK PI3K AKT TSC mTOR Raptor SIN1 S6K S6
Standard Diet
Proteomic data
Model fit
Western Diet
Proteomic data
Model fit
C
mTORC1 Hallmark Genes
129 genes
18 samples
z-score
3
2
1
0
-1
-2
-3
Western Diet
Standard Diet

© The Author(s)

Figure EV3. Absolute quantification of AKT and model-based estimations of total protein abundance.

(**A**) Absolute number of molecules of AKT per primary mouse hepatocyte was determined by quantitative immunoblotting. Based on a dilution curve of recombinant AKT, the number of molecules of AKT in 1 µg lysate was determined. This value was converted with the total protein content per primary mouse hepatocyte into the number of molecules of AKT per cell. (**B**) Measurements of protein abundances derived from primary mouse hepatocytes were implemented in model calibration. Lysates of unstimulated hepatocytes were subjected to data-independent mass spectrometry analysis. Resulting data was LFQ normalized and represented as boxplot: center line indicates median; box limits indicate 25th to 75th percentiles. The lower and upper whiskers extend from the hinge to the smallest or largest value at most 1.5× interquartile range of the hinge. Dots represent the model fit ($n = 9$ per diet). (**C**) A list of Hallmark mTORC1 signaling genes was downloaded from Gene Set Enrichment Analysis (GSEA) and used to filter full proteomes of SD and WD mice. Out of 200 listed proteins, 129 were quantified in all samples and used to cluster samples based on protein abundance using the R package pheatmap.

© The Author(s)

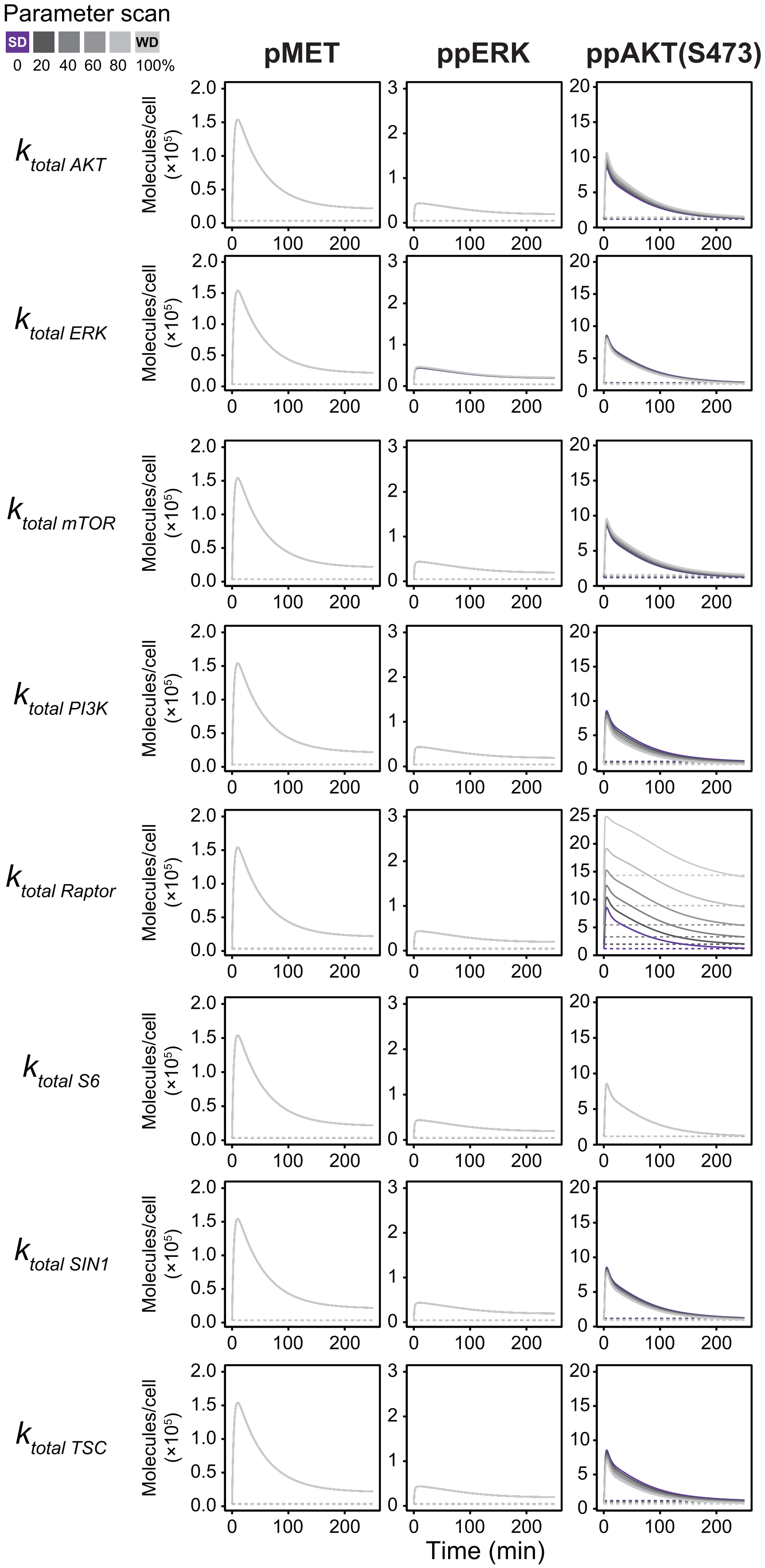
Parameter scan
SD
WD
0 20 40 60 80 100%
pMET
ppERK
ppAKT(S473)
k total AKT
k total ERK
k total mTOR
k total PI3K
k total Raptor
k total S6
k total SIN1
k total TSC
Molecules/cell (×10⁵)
Time (min)

 © The Author(s)

Figure EV4. Influence of dysregulated parameters on protein dynamics.

Individual parameter scan of one dysregulated parameter at a time as explained in Fig. 4A. The value for the indicated parameter was gradually shifted from the SD estimate (purple) to the WD estimate (light gray). The model simulations for the phosphorylation dynamics of MET, ERK and AKT are displayed in molecules/cell. Solid lines indicate model trajectories after HGF stimulation and dashed lines indicate basal levels.

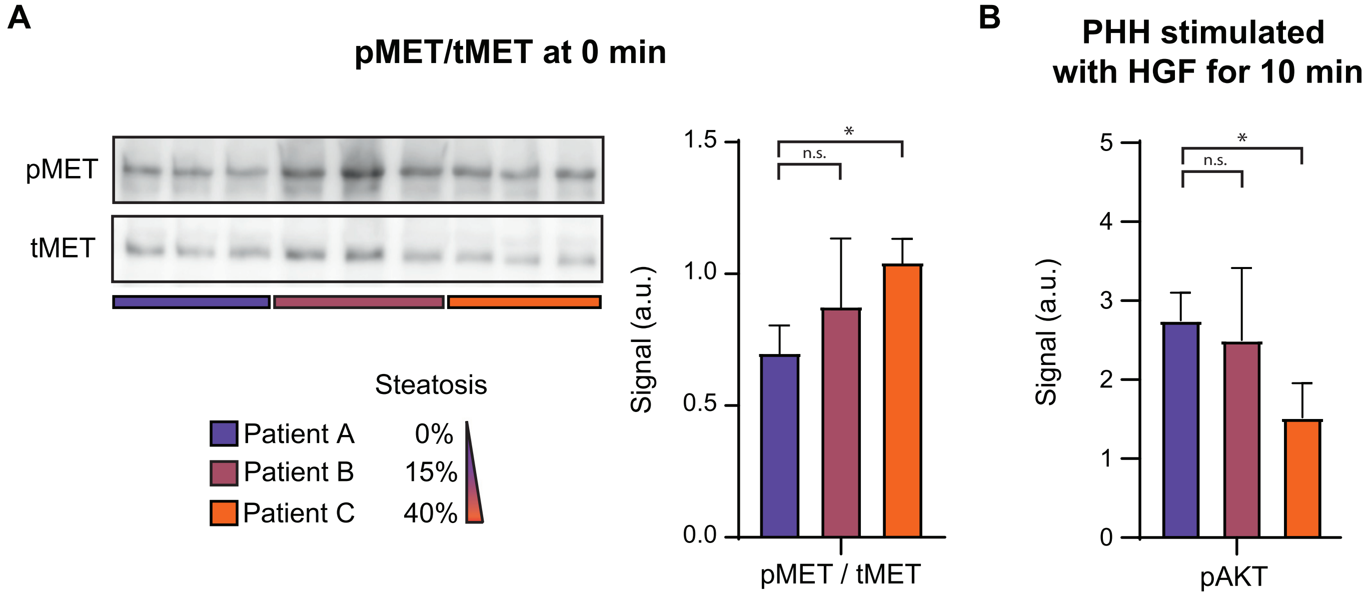

Figure EV5. Signal transduction in primary human hepatocytes from steatotic patients.

(**A**) Isolated primary human hepatocytes from patients with different levels of steatosis were analyzed using quantitative immunoblotting. The ratio of basal MET phosphorylation to MET abundance without HGF stimulation was quantified. Error bars represent one standard deviation ($n = 3$). (**B**) Primary human hepatocytes from patients with different levels of steatosis were stimulated with 40 ng/ml HGF. Phosphorylation of AKT was quantified by immunoblotting after 10 min. p values were calculated using a two-tailed t test (pMET/tMET *0.011, AKT *0.018). Error bars represent one standard deviation ($n = 3$).

 © The Author(s)

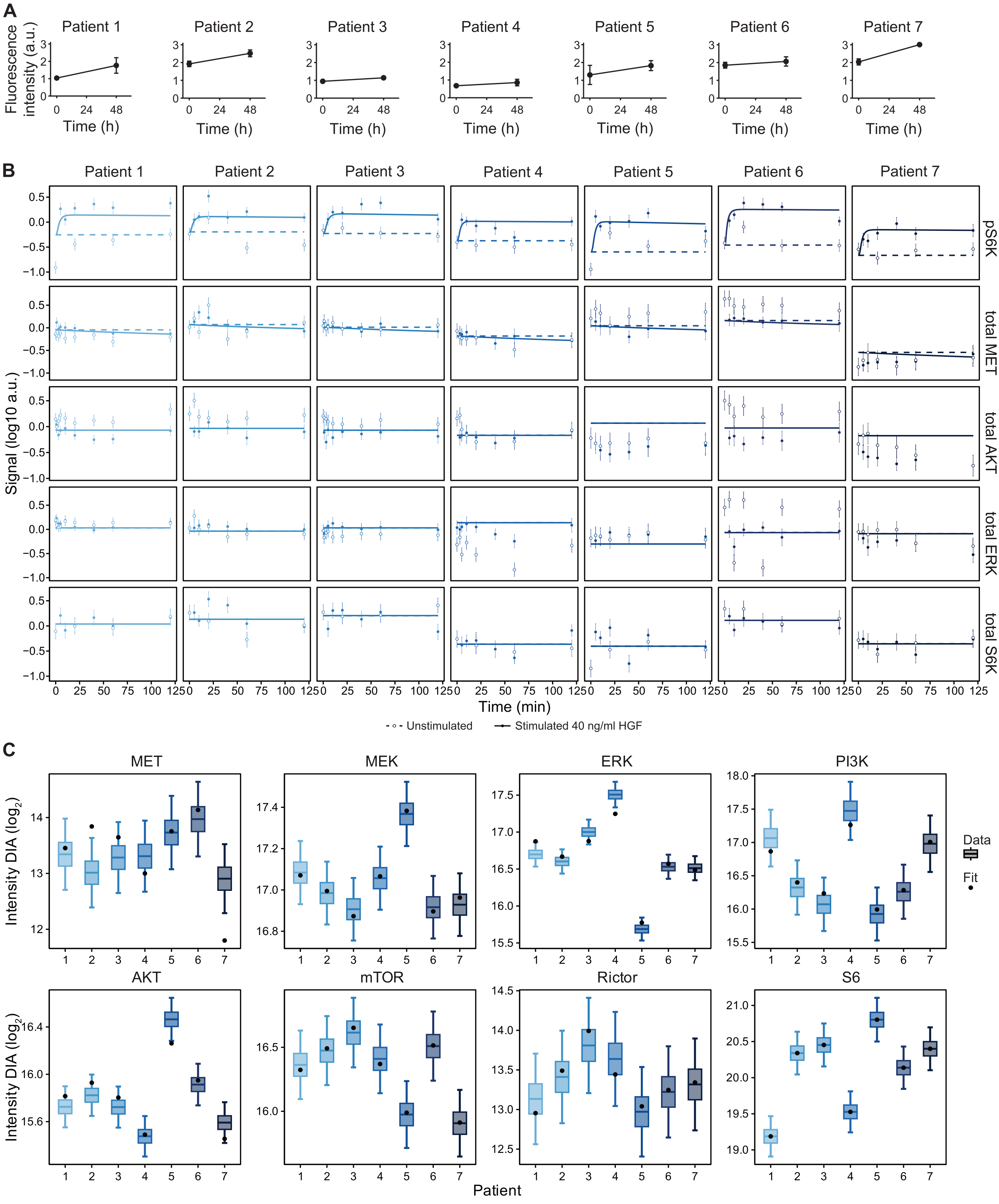
A
Patient 1
Patient 2
Patient 3
Patient 4
Patient 5
Patient 6
Patient 7
Fluorescence intensity (a.u.)
Time (h)
B
pS6K
total MET
total AKT
total ERK
total S6K
Signal (log10 a.u.)
Time (min)
Unstimulated
Stimulated 40 ng/ml HGF
C
MET
MEK
ERK
PI3K
AKT
mTOR
Rictor
S6
Intensity DIA (log2)
Patient
Data
Fit

Figure EV6. Quantitative data for calibration of the human model.

(**A**) Proliferation measurements in isolated primary human hepatocytes from patients. Cells were stimulated with 40 ng/ml HGF. DNA content was measured at time point 0 h and after 48 h by staining with SYBRGreen I. (**B**) Time-resolved immunoblot measurements and model fits for pS6K as well as MET, AKT, ERK and S6K abundance in primary human hepatocytes derived from seven patients. Cells were stimulated with 40 ng/ml HGF or left untreated. Signal is shown in $\log_{10}$ arbitrary units (a.u.). Data points are displayed as dots along with error bars representing 1σ confidence interval estimated from technical replicates ($n = 1$–3 per patient) using a combined scaling and error model. Model trajectories are depicted as lines. (**C**) Measurements for protein abundances derived from primary patient hepatocytes were included as additional data for model calibration. Lysates of unstimulated hepatocytes were subjected to data-independent mass spectrometry analysis ($n = 1$–3 per patient). Resulting data was normalized using label-free quantification and represented as boxplot: center line indicates the patient median; box limits are defined as 1σ, calculated based on the mean spread of the cohort per protein. The lower and upper whiskers extend from the center line by 3σ. Model fits are represented as black dots.

© The Author(s)
